# Playing both sides – Epstein-Barr Virus accumulates select cellular transcripts to counter virus-mediated host shut-off in lytic cells

**DOI:** 10.1371/journal.ppat.1014211

**Published:** 2026-05-11

**Authors:** Derek Daigle, Ashton Creasy-Marrazzo, Lyn Gradoville, Ayman El-Guindy, Rishabh Mukherjee, Sandeepan Das, Budhadev Baral, Beth A. Rousseau, Richard Park, Michael T. McIntosh, George Miller, Sumita Bhaduri-McIntosh

**Affiliations:** 1 Molecular Biophysics and Biochemistry, Yale University School of Medicine, New Haven, Connecticut, United States of America; 2 Pediatrics, University of Florida, Gainesville, Florida, United States of America; 3 Pediatrics, Yale University School of Medicine, New Haven, Connecticut, United States of America; 4 Epidemiology and Public Health, Yale University School of Medicine, New Haven, Connecticut, United States of America; 5 Molecular Genetics and Microbiology, University of Florida, Gainesville, Florida, United States of America; 6 AbVir Biotherapeutics, San Diego, California, United States of America; University of Utah, UNITED STATES OF AMERICA

## Abstract

Epstein-Barr virus (EBV), an oncogenic virus, actively remodels the intracellular environment during its lytic (replicative/productive) phase to facilitate genome replication, virion packaging, and egress while attempting to evade immune responses. A key aspect of this remodeling is the downregulation of host gene expression, a phenomenon known as host shutoff. This process is prominently mediated by the EBV-encoded nuclease *BGLF5*, but *BGLF5*-independent mechanisms also contribute – most notably, the viral lytic switch protein ZEBRA, which can suppress host protein synthesis. Despite this broad suppression, the expression of certain host genes essential for lytic progression must be preserved or even enhanced. To investigate how such genes evade host shutoff, we analyzed the expression of a set of cellular transcripts in Burkitt lymphoma cells, sorted 24 hours after exposure to lytic triggers, into lytic and refractory (non-lytic) populations. We identified a subset of host transcripts consistently upregulated in lytic cells across Burkitt lymphoma lines reactivated by functionally distinct lytic stimuli, indicating that such upregulation is independent of pleiotropic lytic cycle inducing stimuli. Importantly, we found that while ZEBRA suppresses protein expression of some of these host (and select viral) genes, EBV also transcriptionally upregulates two related host genes, *ELAVL4* and *PABPC4L*. Both encode RNA-binding proteins, and we found that they differentially modulate viral transcript abundance – enhancing some while repressing others – ultimately supporting the transcriptional demands, efficient genome replication and virion release during the EBV lytic cycle. These findings highlight the virus’s finely tuned regulation of both host and viral gene expression to ensure successful completion of the lytic cycle. Specifically, they suggest that EBV selectively upregulates critical host genes to counteract/escape host shutoff and promote virus propagation.

## Author summary

During its lytic phase, Epstein-Barr virus (EBV) reprograms the host cell environment to support viral replication, packaging, and egress while evading immune responses. A key strategy is virus-mediated host shutoff, largely driven by the EBV *BGLF5*-encoded nuclease and the lytic switch protein ZEBRA, both of which suppress host gene expression. To understand how certain host genes critical for the lytic phase escape shutoff, we analyzed cellular transcripts in Burkitt lymphoma cells sorted into lytic and non-lytic populations. We identified a subset of transcripts consistently and selectively upregulated in lytic cells, regardless of the lytic trigger or cell line. Importantly, while ZEBRA suppresses select host and viral proteins, EBV also transcriptionally activates host genes such as *ELAVL4* and *PABPC4L* – these encode two RNA-binding proteins that we find regulate the abundance of lytic transcripts and proteins, promoting EBV genome replication and virion release. These findings reveal that EBV fine-tunes host and viral gene expression during the lytic cycle, using ZEBRA to selectively upregulate critical host gene transcription in lytic cells to offset host shutoff, ensuring completion of the lytic phase.

## Introduction

Epstein-Barr virus (EBV) establishes lifelong latent infection in the vast majority of the global adult population. Although largely asymptomatic during latency, EBV was implicated in approximately 300,000 cancer cases and nearly 200,000 cancer-related deaths in 2020 [1]. A critical aspect of EBV’s long-term persistence is its ability to periodically reactivate into the lytic cycle, facilitating viral transmission. Importantly, while tumors arise from latently infected cells, lytic reactivation plays an important role in oncogenesis. To avoid immune recognition during the lytic phase, EBV co-opts host cellular pathways and employs a range of mechanisms to subvert both innate and adaptive immune responses [2]. Central to this strategy is the virus’s induction of global suppression of host gene expression – known as “host shutoff” – which is initiated during the early stages of the lytic cycle. This shutoff not only facilitates efficient transcription and replication of the viral genome while minimizing inflammatory and immune detection, but also redirects cellular resources toward virus production.

In gammaherpesviruses such as EBV and Kaposi’s Sarcoma-associated Herpesvirus (KSHV), host shutoff is classically mediated by lytic gene products. Chief among these are the EBV BGLF5 protein and the KSHV SOX protein, which, in addition to their roles as DNA alkaline exonucleases, promote host shutoff by destabilizing cellular mRNAs [3]. However, BGLF5-independent mechanisms – distinct from RNA degradation – also contribute significantly to this process. Previously, we demonstrated that the EBV lytic switch protein ZEBRA can suppress host protein synthesis by inhibiting translation and causing relocalization of PABPC (Poly(A)-binding protein, cytoplasmic) into the nucleus [4]. More recently, using nascent transcriptomic analysis, we also showed that transcriptional downregulation of host genes occurs both upstream and downstream of ZEBRA expression during the EBV lytic phase [5]. Consistent with these findings, a subsequent study revealed impaired host gene transcription and RNA processing in the absence of BGLF5, further underscoring the existence of BGLF5-independent pathways of host shutoff [6]. However, the expression of select host genes must be preserved, or even upregulated, in order to sustain essential cellular functions that support viral gene expression, genome replication, and the assembly and release of progeny virions. To better understand this balance, we sought to identify host transcripts that are upregulated during the EBV lytic phase, potentially as a preemptive or compensatory response to virus-mediated host shutoff.

Detailed molecular studies of the EBV lytic cycle are made possible by the use of cultured cell lines harboring the virus in a latent state. Treatment of EBV-positive Burkitt lymphoma cell lines with a variety of stimuli, including, but not limited to, histone deacetylase inhibitors (HDACi), DNA methyl transferase inhibitors, protein kinase C agonists, or anti-IgG immunoglobulin promotes reactivation of the viral lytic cycle [7–11]. One complicating factor in interpreting data from such experiments is that only a fraction of the cellular population is triggered into the lytic cycle by these inducing stimuli. Depending on the cell line and the stimulus, 5% to 40% of cells express viral lytic genes. The virus remains latent in the remainder of the treated population that is refractory to viral reactivation [9,12–14]. Thus, studies attempting to correlate cellular gene expression with viral reactivation, or conversely, the effects of the viral lytic cycle on expression of cellular genes, must deal with a large background of cellular gene expression in the majority of cells not supporting reactivation. To overcome this issue, we frequently use the Burkitt lymphoma cell line HH514–16 in which EBV is tightly latent at baseline but can be readily triggered into the lytic phase by HDACi such as sodium butyrate (NaB) and trichostatin A (TSA), or by the DNA methyltransferase inhibitor azacytidine (AzaCdR) [8,15]. HH514–16 was cloned from the P3J-HR1K cell line that, in turn, was derived from the Jijoye Burkitt lymphoma cell line [16]. Following lytic cycle induction with different stimuli, we use a FACS-based technique to efficiently separate subpopulations of HH514–16 cells that are refractory or responsive to lytic induction [13,14,17,18]. Cell separation enables detection of changes in cellular gene expression that occur within each subpopulation relative to each other or to untreated cells. This technique also removes much of the background cellular gene expression that each population contributes to the other when a mixture of unsorted cells is studied.

To understand how the host contributes to a successful lytic phase despite virus-mediated host shutoff, we used sorted HH514–16 cells to identify a set of cellular transcripts that were upregulated selectively in lytic cells following exposure to functionally diverse lytic stimuli. Of these, transcripts encoding two RNA-binding proteins, ELAVL4/HuD and PABPC4L, were also upregulated in lytically activated Akata Burkitt lymphoma cells. Both *ELAVL4* and *PABPC4L* were transcriptionally activated by introduction of ZEBRA, though only in EBV-infected cells, to counter ZEBRA-mediated shutoff of ELAVL4 and PABPC4L proteins. Of note, we found that ZEBRA also selectively shut off viral proteins such as EA-D (*BMRF1* gene product), thereby tightly regulating the lytic cycle. Importantly, upregulation of *ELAVL4* and *PABPC4L* mRNA abundance offset ZEBRA-mediated shutoff of ELAVL4 and PABPC4L protein expression. We show that this process of modulating the abundance of select cellular transcripts in lytic cells is required to effectively support EBV genome replication and virion release.

## Results

### Distinct cellular gene expression patterns characterize refractory and lytic subpopulations

**Fig 1:**
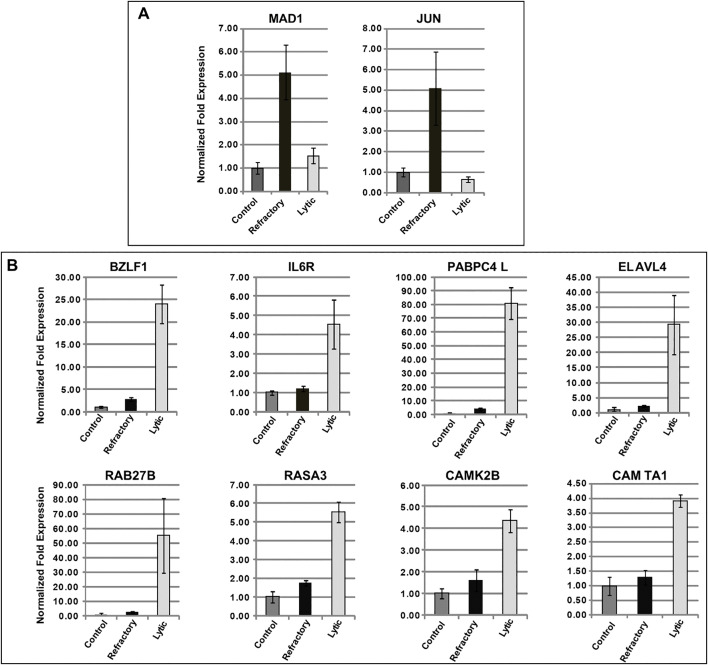
Selective up-regulation of cellular genes in refractory and lytic subpopulations of HH514-16 cells treated with NaB. HH514-16 cells were untreated (control) or treated with NaB for 48 hours. The cells treated with NaB were sorted into lytic and refractory subpopulations. Control cells were mock sorted. Total RNA was isolated from each population. Levels of mRNA were measured by RT-qPCR using gene-specific primers. **(A)**. Data are shown for *MAD1* and *JUN*, genes that were preferentially expressed in the refractory population. **(B)**. Levels of mRNA are shown for *BZLF1*, *IL6R*, *PABPC4L*, *ELAVL4*, *RAB27B*, *RASA3*, *CAMK2B*, and *CAMTA1*, genes that were selectively expressed in the lytic population. Error bars represent SD from three technical replicates.

We previously reported that expression of several cellular genes, e.g., *STAT3*, *FOS*, and genes that encode components of the constitutive heterochromatin machinery including three members of the KRAB-ZFP family (*SZF1/ZNF589*, *ZNF557*, *ZNF253*) and the histone methyl transferase *SETDB1*, is up-regulated only in HH514–16 cells that are refractory to NaB-mediated lytic induction of EBV [14,17]. We also showed that the viral host cell shutoff mechanism, which is expected to function in the lytic, but not in the refractory subpopulation, does not prohibit increases in expression of all cellular genes; for example, IL6 transcript levels were increased preferentially in the lytic cell population as were transcripts of the inflammasome intermediary protein TXNIP [14,19]. To extend these observations, we sorted HH514–16 cells into refractory and lytic subpopulations 24 hours after treatment with NaB (as shown in S1 Fig) and compared cellular gene expression in each subpopulation with cells that had not been treated with NaB. We first examined the abundance of transcripts of a subset of cellular genes that we previously found were upregulated in the total cell population after treatment with the HDAC inhibitors NaB or TSA [14]. Following exposure to NaB, we observed increased levels of *MAD1* and *JUN* transcripts in sorted refractory relative to untreated or sorted lytic cells (Fig 1A), adding to the set of cellular transcripts we had previously found upregulated in refractory cells [14,17]. Thus, although microarray analyses of total HH514–16 cell populations treated with NaB and TSA identified increases and decreases in expression of a large number of cellular genes [14], increases in transcript abundance of some of these genes occurred selectively in the subpopulation of cells that was refractory to lytic viral activation.

**Fig 2:**
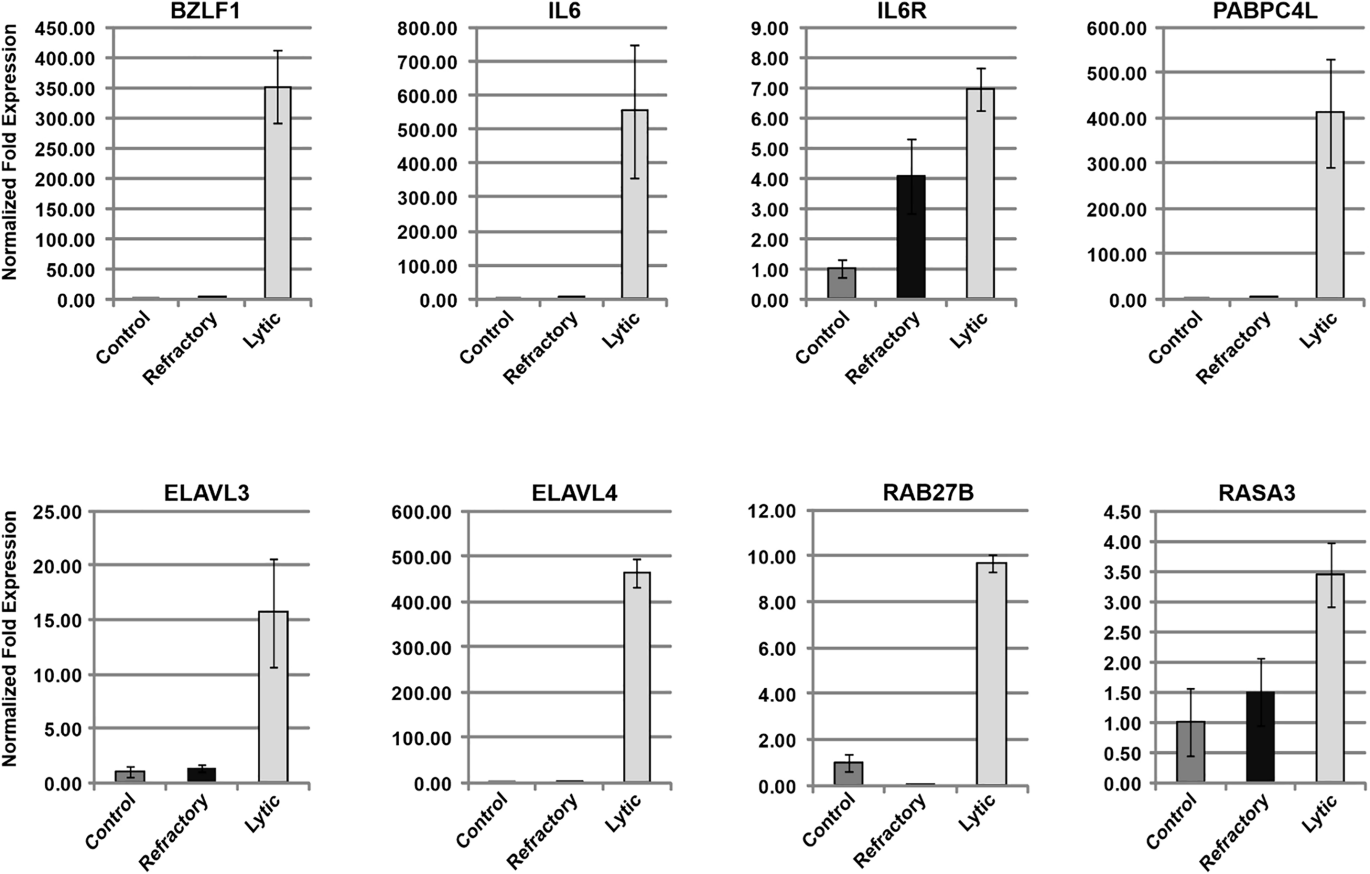
Examples of cellular genes whose expression is upregulated in lytic HH514-16 cells following treatment with AzaCdR. HH514-16 cells were untreated or treated with the DNA methyltransferase inhibitor 5-aza-2’-deoxycytidine (AzaCdR) for 48 hours. The cells were sorted into lytic and refractory subpopulations as described in S1 Fig and total RNA extracted. Transcripts were quantified by RT-qPCR relative to untreated control using gene-specific primers. Data is shown for *BZLF1*, *IL6*, *IL6R*, *PABPC4L*, *ELAVL3*, *ELAVL4*, *RAB27B*, and *RASA3*. Error bars represent SD from three technical replicates.

As a preliminary guide to search for genes whose transcripts were preferentially upregulated in lytic cells, we examined microarrays that profiled gene expression in sorted cells [17]. Examples of a subset of genes whose expression was increased in the lytic population based on the microarray [17] are listed in Table 1. These genes were selected for validation and further study because of their potential biological properties. For example, cellular genes induced in the lytic sub-population included the IL6 cytokine that might have anti-apoptotic function in B cells, PABPC4L and ELAVL3 and ELAVL4, genes that might affect mRNA biogenesis or stability, and several genes that might specifically alter cell signal transduction. Examination of transcript abundance showed that as expected, *BZLF1* transcripts were ~10 fold more abundant in NaB-exposed/sorted lytic compared to refractory and untreated control HH514–16 cells (Fig 1B). Also, as expected, the shut-off protein BGLF5 was observed almost exclusively in sorted lytic cells compared to sorted refractory and untreated cells (S2 Fig). As for cellular transcripts, we found that like IL6, whose upregulation we previously validated in sorted lytic cells [14], the remaining candidate genes from Table 1 all exhibited increased expression in lytic relative to untreated or refractory cells (Fig 2). Notably, based on the relative abundance of transcripts in lytic cells 24 hours after treatment with NaB, these genes fell into two groups: transcripts of *PABPC4L*, *ELAVL4* and *RAB27B*, were markedly increased at 30–80-fold, whereas the expression of the other four genes increased 4- to 5-fold in the lytic population. Thus, several cellular transcripts demonstrated selective upregulation in lytic cells.

**Table 1:** Examples of cellular genes whose expression was preferentially up-regulated in lytic HH514-16 cells relative to untreated or refractory cells based on microarray analysis. HH514-16 cells were untreated or treated with NaB for 24 hours and sorted into lytic and refractory subpopulations. Total RNA was extracted from each subpopulation and analyzed on Affymetrix U133 Plus 2.0 arrays (published in Hill et al. [17]).

Gene	Name	Fold up-regulation in lytic cells^a)^
*IL6*	Interleukin 6	2.3
*IL6R*	Interleukin 6 Receptor	12.1
*PABPC4L*	Poly A binding protein cytoplasmic 4-like	9.2
*ELAVL3*	Embryonic lethal abnormal vision-like 3	32.0
*ELAVL4*	Embryonic lethal abnormal vision-like 4	3.5
*RAB27B*	RAB27B, member RAS oncogene family	A to P^b)^
*RASA3*	RAS p21 protein activator 3	104
*CAMK2B*	calcium/calmodulin-dependent protein kinase (CaM kinase) II beta	18.4
*CAMTA1*	calmodulin binding transcription activator 1	13.0

a) For all these genes there was no change in the refractory population, by comparison to the control population.

b) A to P: absent to present; it was not possible to calculate the fold-change in the lytic population for this gene.

### Upregulation of cellular transcripts in the lytic subpopulation of HH514–16 cells is also observed after exposure to a mechanistically distinct lytic stimulus

One drawback of using HDAC inhibitors to induce the EBV lytic cycle is that these agents mediate extensive changes in cellular gene expression [5,14,20–23]. Therefore, we needed to consider that the observed increases in cellular transcripts in lytic cells did not correlate with viral activation but were at least partially due to direct effects of HDAC inhibition on cellular gene expression. An advantage of using HH514–16 cells for studying EBV reactivation is that two different classes of stimuli with distinct mechanisms of action induce the viral lytic cycle. Treatment of this cell line with AzaCdR, a DNA methyltransferase inhibitor, also activates the EBV lytic cycle. By comparison to HDAC inhibitors, AzaCdR promotes only slight changes in cellular gene expression [23]. To determine if the cellular transcripts observed to accumulate in lytic cells after NaB treatment were also upregulated in AzaCdR-induced lytic cells, HH514–16 cells were untreated or treated with AzaCdR for 48h and sorted into refractory and lytic subpopulations. RT-qPCR analysis showed that *BZLF1* transcripts were markedly upregulated in lytic cells by comparison to refractory cells, indicating that the two subpopulations were separated efficiently (Fig 2). Six genes whose expression was increased in the lytic subpopulation of HH514–16 cells treated with NaB were also found to be upregulated in lytic cells following treatment with AzaCdR; these included *IL6*, *PABPC4L*, *ELAVL3*, *ELAVL4*, *RAB27B* and *RASA3*. Furthermore, as in NaB-induced sorted lytic cells (Fig 1B), *PABPC4L*, *ELAVL4* and *RAB27B* transcripts demonstrated the highest fold elevation in lytic compared to refractory cells (Fig 2). As for *IL6R*, although transcripts were increased as in NaB-induced lytic cells (Fig 1B), they were also elevated in the refractory subpopulation relative to untreated cells (Fig 2). These results demonstrate that upregulation of a subset of cellular genes in HH514–16 cells is tightly correlated with activation of EBV lytic gene expression, and is observed with two lytic cycle inducing stimuli with different modes of action.

### Valproic acid inhibits expression of cellular genes characteristic of lytic HH514–16 cells

**Fig 3:**
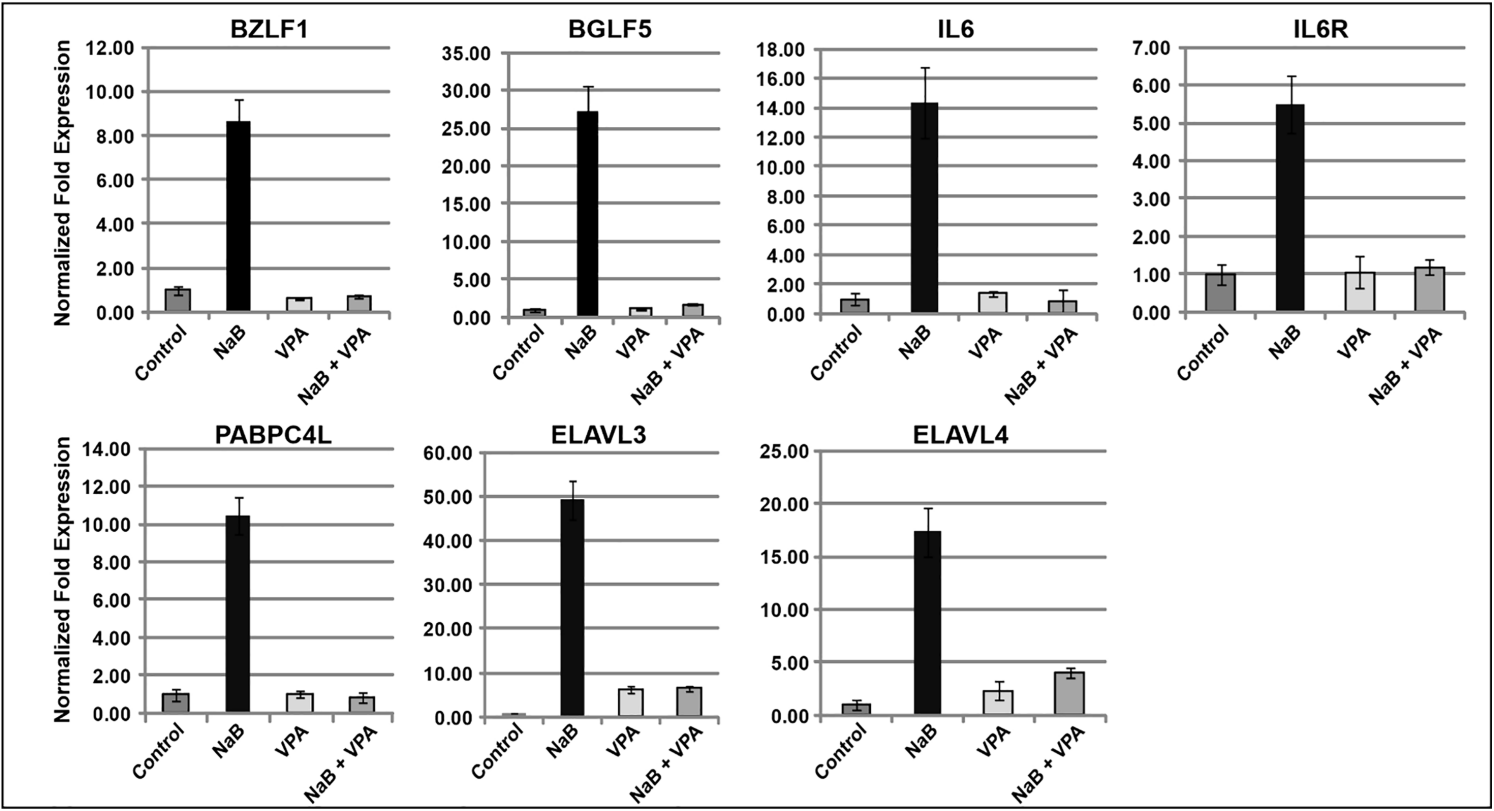
Valproic acid suppresses expression of cellular genes characteristic of the lytic cellular subpopulation. HH514-16 cells were untreated or treated with NaB, VPA, or NaB and VPA, harvested after 24 hours, and total RNA extracted. Levels of mRNA were measured by RT-qPCR relative to untreated control using gene-specific primers. Data is shown for *BZLF1*, *BGLF5*, *IL6*, *IL6R*, *ELAVL3*, *ELAVL4*, and *PABPC4L*, genes whose expression is upregulated in lytic cells. Data are representative of two biological replicates; error bars represent SD from three technical replicates.

We previously reported that in HH514–16 cells, the HDAC inhibitor valproic acid (VPA) antagonizes the lytic cycle-inducing activity of other stimuli, including other HDAC inhibitors and AzaCdR [23]. However, in this cell line VPA activates and suppresses expression of many cellular genes in a manner generally similar to other HDACi, such as NaB and TSA [24]. These findings offered the opportunity to further clarify the correlation of our candidate cellular genes with EBV lytic cycle activation. HH514–16 cells were untreated (control) or treated with NaB, VPA, or NaB combined with VPA for 24 hours and total RNA extracted. As expected, treatment with NaB activated *BZLF1* and *BGLF5* expression; however, as we had previously shown, treatment with VPA failed to activate expression of these EBV lytic genes, and combining VPA with NaB completely inhibited expression of *BZLF1* and *BGLF5* (Fig 3). We investigated the expression of five cellular genes, *IL6*, *IL6R*, *PABPC4L ELAVL3*, and, *ELAVL4*, which were induced in lytic HH514–16 cells after NaB or AzaCdR treatment. Expression of all five cellular genes was increased following treatment with NaB, while no significant increases were observed following VPA treatment (Fig 3). The combination of VPA with NaB completely inhibited the increases in expression of all five cellular genes that were observed following NaB treatment alone. In contrast, we had previously observed that treatment of HH514–16 cells with VPA or VPA plus NaB increased expression of cellular genes, such as *STAT3*, *FOS*, *FRMD6*, *SEPP1* and *MAD1* in cells refractory to lytic activation by NaB [23]. These results are consistent with the conclusion that expression of one subset of cellular genes is tightly associated with EBV lytic cycle activation whereas a different subset of cellular transcripts is upregulated in cells that fail to respond to lytic cycle inducing stimuli.

### Expression of cellular genes characteristic of lytic cells is induced in EBV(+), but not in EBV(-) Akata cells treated with anti-IgG

**Fig 4:**
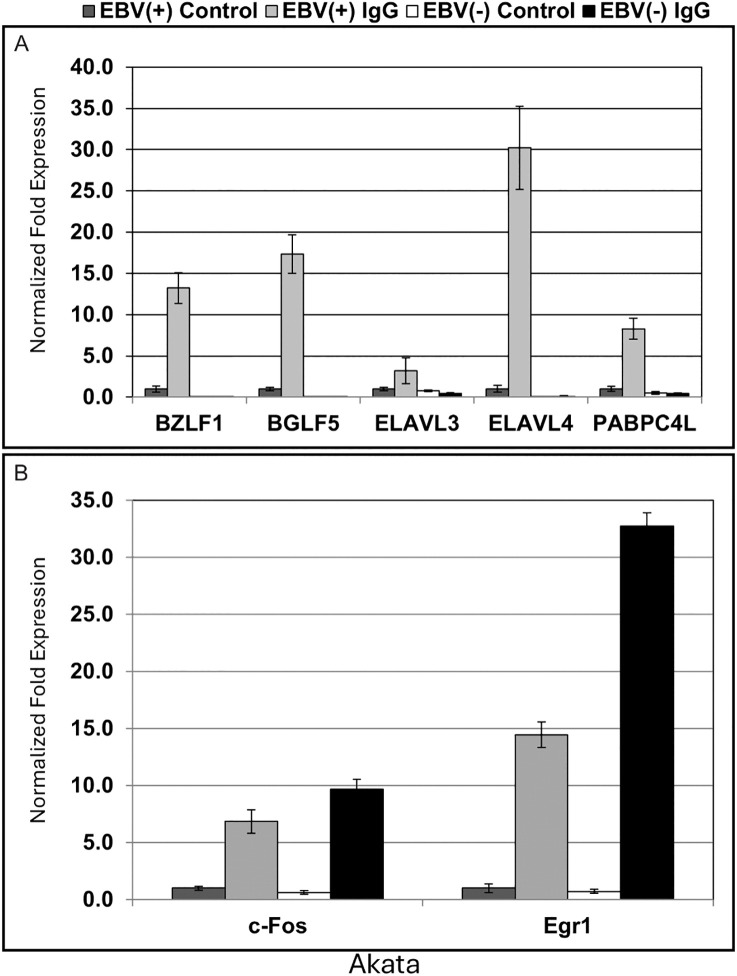
Comparison of cellular gene expression in EBV-positive and EBV-negative Akata cells following treatment with anti-IgG. EBV(+) Akata or EBV(-) Akata cells were untreated or treated with anti-IgG, harvested after 24 hours, and total RNA extracted. Transcripts were quantified by RT-qPCR relative to untreated controls and data are shown for **(A)** *BZLF1*, *BGLF5*, *PABPC4L*, *ELAVL4*, and *ELAVL3* (genes whose expression is upregulated in lytic HH514-16 cells; Figs 1 and 2) and **(B)** *FOS* and *EGR1*, cellular immediate-early genes. Data are representative of two biological replicates; error bars represent SD from three technical replicates.

The foregoing results demonstrated a strong association between expression of several cellular genes and the EBV lytic cycle that was independent of the inducing stimulus. To assess generalizability beyond the HH514–16 cell line, we evaluated the expression of a subset of these genes, i.e., three RNA biogenesis-related cellular genes that mark lytic cells, in a different cellular background. The EBV-positive (+) Akata cell line derived from a Burkitt lymphoma can be activated into the lytic cycle by cross-linking the B-cell antigen receptor with anti-IgG. For comparison we also measured cellular gene expression in a derivative EBV-negative (-) Akata cell line from which the EBV genome has been lost. EBV(+) and EBV(-) Akata cells were untreated or treated with anti-IgG, harvested after 24 hours, and total RNA extracted. Expression of viral and cellular genes was analyzed by RT-qPCR relative to untreated controls. As expected, expression of the EBV early lytic transcripts *BZLF1* and *BGLF5* was observed in EBV(+), but not EBV(-), Akata cells following anti-IgG treatment (Fig 4A). Of the three genes tested, increased levels of transcripts of the cellular genes *ELAVL4* and *PABPC4L* were detected in EBV(+) Akata cells after treatment with anti-IgG. Under the same conditions there was no increase in expression of these genes in EBV(-) Akata cells (Fig 4A). A slight increase in *ELAVL3* transcripts was also observed in EBV(+) Akata cells, but was not as robust as the increases in *ELAVL4* and *PABPC4L* transcripts. Of note, EBV(-) Akata cells did respond to anti-IgG treatment; transcripts of two cellular immediate-early genes, *FOS* and *EGR1* [23], were elevated in response to anti-IgG treatment in both EBV(+) and EBV(-) Akata cells relative to untreated controls (Fig 4B). These results reinforce the conclusion that increased expression of some cellular genes is specifically associated with EBV lytic cycle induction.

### Expression of the cellular genes *ELAVL4* and *PABPC4L* in lytic HH514–16 cells is kinetically downstream of EBV early lytic gene expression

The foregoing experiments identified a subset of cellular genes whose expression was preferentially stimulated within those HH514–16 or Akata cells in which the EBV lytic cycle was induced. Since these observations were made at a single time point, 24–48 hours after cells were treated with inducing stimuli, we could not establish from these experiments whether the changes in expression of the cellular genes were upstream or downstream of viral lytic activation. To determine when the increases in cellular gene expression occurred relative to increases in viral lytic gene expression, total RNA was extracted from HH514–16 cells that were untreated or treated with TSA or AzaCdR and harvested at 8, 12, 18, 24, or 48 hours. Levels of RNA for *BZLF1*, *BGLF5*, and two marker cellular genes, *ELAVL4* and *PABPC4L*, whose expression was robustly stimulated in lytic cells after NaB and AzaCdR treatment (Figs 1B and 2), were measured by RT-qPCR at each time (Table 2). Previous experiments have shown that *BZLF1* transcripts can be detected by 8 hours following treatment with NaB or AzaCdR [8,25]. In this experiment a low-level induction of *BZLF1* and *BGLF5* mRNA was observed by 8 hours following treatment with either TSA or AzaCdR; robust activation of the viral lytic mRNAs was observed at 12 hours. Increases in expression of *BZLF1* reached a maximum level of approximately 35-fold the background of untreated cells at 18 hours after treatment with TSA, and about 22-fold at 24 hours after exposure to AzaCdR. By contrast to the lytic viral mRNAs, no increases in *ELAVL4* and *PABPC4L* transcript levels above background were detected at 8 hours after exposure to either lytic cycle inducing agent. At 12 hours, *ELAVL4* expression was elevated 5.8-fold in cells treated with TSA, but *PABPC4L* was at background levels; at this time neither cellular gene was expressed above background levels in cells exposed to AzaCdR. The maximum levels of expression of these two cellular genes was delayed to 24 or 48 hours after addition of the inducing stimulus.

**Table 2:** Expression of two cellular genes induced in lytic HH514-16 cells is kinetically downstream of the EBV early lytic transcripts *BZLF1* and *BGLF5*. Total RNA was extracted from HH514-16 cells harvested 8, 12, 18, 24, or 48 hours following treatment with TSA or AzaCdR. RT-qPCR data for *BZLF1*, *BGLF5*, *ELAVL4*, and *PABPC4L* are shown relative to untreated controls. The value is shown in bold and circled at the time when the expression level of the gene first exceeded the background by 2-fold.

HH514–16 Cells Treated with TSA					
	8h	12h	18h	24h	48h
***BZLF1***	**4.2**	28.2	35.5	34.1	35.3
***BGLF5***	**2.9**	104.8	88.9	168.3	234.3
***ELAVL4***	1.1	**5.8**	7.5	44.6	82.0
***PABPC4L***	1.2	1.1	**5.8**	50.1	95.2
**HH514–16 Cells Treated with AzaCdR**					
	**8h**	**12h**	**18h**	**24h**	**48h**
***BZLF1***	**2.6**	7.9	5.9	22.3	22.9
***BGLF5***	**2.8**	16.8	18.5	63.6	103.3
***ELAVL4***	0.9	1.0	**2.9**	22.1	21.3
***PABPC4L***	0.7	1.5	**19.9**	109.7	58.8

Since steady state RNA levels do not distinguish between altered transcription rates and RNA stability, we examined a previously published nascent transcriptomic dataset [5] for evidence of transcriptional upregulation of *ELAVL4* and *PABPC4L*. In those experiments, we mapped nascent transcripts by exposing HH514–16 cells to NaB for 3 hours (temporally upstream of *BZLF1* expression) as well as 24 and 48 hours (temporally downstream of *BZLF1* expression) and subjected them to Bru-Seq analysis. In Bru-Seq, RNA is tagged during synthesis with a 30-minute pulse of bromouridine (BrU) prior to harvest, followed by immuno-separation of tagged nascent RNA from total RNA using anti-BrdU antibodies, and sequencing of fragmented cDNA strands from reverse-transcribed purified nascent transcripts. For additional details, please see Frey et al. [5]. Normalized reads of nascent transcripts shown in Table 3 indicate that there was no transcription of *ELAVL4* or *PABPC4L* at baseline or after 3 hours of exposure to NaB. However, consistent with our observations on steady state transcripts in Table 2, we noted transcriptional upregulation of both genes in both experimental replicates by 24 hours (~60–185 for *ELAVL4* and 45–65 for *PABPC4L*) with no further increase at 48 hours. These results demonstrate that transcriptional upregulation of these two cellular genes that are induced specifically in HH514–16 cells in which the EBV lytic cycle has been activated, are kinetically downstream of the expression of EBV early lytic genes.

**Table 3:** *ELAVL4* and *PABPC4L* are transcriptionally upregulated by 24 hours following lytic cycle reactivation. Normalized reads of nascent *ELAVL4* and *PABPC4L* transcripts from two replicates of Bru-seq datasets derived from HH514-16 cells exposed to NaB for 3, 24, and 48 hours (Frey et al. *The Journal of Virology* 2020).

	NaB	Norm reads at 3h	Norm reads at 24h	Norm reads at 48h
***ELAVL4***	–	0.8/0	0/1.2	0.7/0
	+	0/0.9	185/61.1	184.1/39.9
***PABPC4L***	–	0/0	0/0	0.7/0
	+	0/0	66/47.3	36.2/25.6

Replicate 1/ Replicate 2

In assessing the relationship to viral genome replication that is temporally downstream of early gene expression, we found that *ELAVL4* and *PABPC4L* transcripts rose in lytically induced HH514–16 cells even when viral genome replication was blocked by phosphonoacetic acid (PAA) (S3A and S3C Fig). In a lymphoblastoid cell line (LCL), lytic cycle induction also resulted in upregulation of both transcripts but PAA prevented the rise of *PABPC4L* transcripts (S3A and S3C Fig). Importantly, the HuD protein, encoded by *ELAVL4*, increased in both cell types regardless of PAA (S3B Fig). As expected, lytic cycle triggers induced the expression of *BZLF1* transcripts (S3D Fig) while PAA suppressed viral genome replication (S3E Fig). Thus, *ELAVL4*/HuD and *PABPC4L* are induced downstream of early EBV genes but upstream of viral genome replication, with *PABPC4L* in LCL partially dependent on genome replication.

### The EBV lytic switch gene *BZLF1* activates expression of *ELAVL4* and *PABPC4L* transcripts selectively in EBV-infected cells

**Fig 5:**
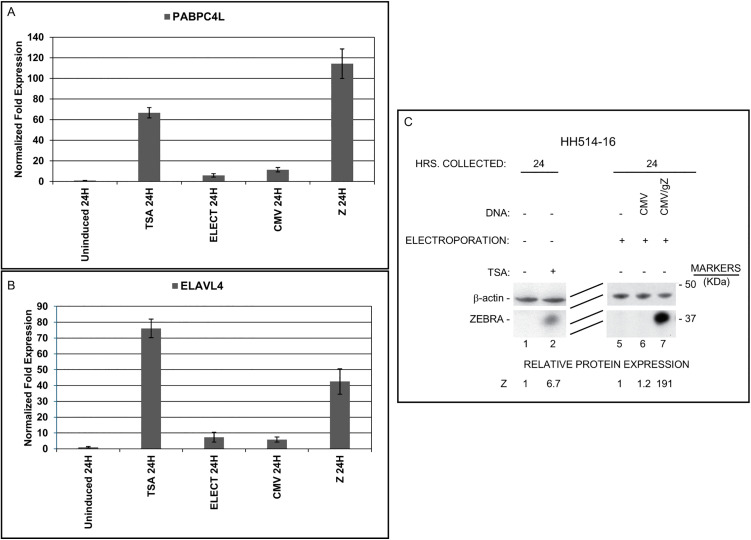
Transfection of a vector expressing the EBV *BZLF1* gene activates cellular genes encoding RNA binding proteins. HH514-16 cells were untreated or treated with TSA. Other aliquots were mock transfected by electroporation or transfected with CMV vector or CMV gZ, encoding *BZLF1*. RNA harvested after 24 hours was analyzed for expression of *PABPC4L* **(A)** or *ELAVL4* **(B)** by RT-qPCR. Parallel samples from the same experiment were analyzed by immunoblotting for expression of ZEBRA protein and β-actin **(C)**. Error bars, SD from three technical triplicates.

Experiments described in Figs 1–4 demonstrated a correlation between expression of *ELAVL4* and *PABPC4L* and EBV lytic cycle activation by a diverse array of lytic stimuli. The experiments in Tables 2 and 3, showing that expression of *ELAVL4* and *PABPC4L* was delayed relative to expression of early lytic genes *BZLF1* and *BGLF5*, suggested that one or more EBV lytic products could be responsible for activating expression of these cellular genes. It was therefore essential to learn whether transcripts encoding the RNA binding proteins could be induced in the absence of pleiotropic stimuli such as HDAC inhibitors, AzaCdR, or anti-IgG. Accordingly, HH514–16 cells were transfected with a plasmid expressing ZEBRA. Cells that were mock electroporated or transfected with empty vector served as controls. In the same experiment, other batches of cells were untreated or treated with TSA which was known to activate these genes (Table 2). Cells were harvested at 24 hours and analyzed for expression of *PABPC4L* or *ELAVL4* mRNAs (Fig 5A and 5B) or ZEBRA protein (Fig 5C). Transfection of *BZLF1* strongly activated *PABPC4L* mRNA (102-fold) and *ELAVL4* mRNA (49-fold). Consistent with the data in Table 2, TSA also strongly activated both genes at 24 hours. Immunoblotting (Fig 5C) confirmed the expression of ZEBRA following electroporation or treatment with TSA.

To address if ZEBRA also induced the expression of *ELAVL4* and *PABPC4L* in EBV-uninfected cells, we introduced *BZLF1* into HH514–16 cells and compared the response in the EBV-negative B lymphoma cell line BJAB. As shown in S4 Fig, although *BZLF1* transcripts were abundantly expressed in both cell lines, *ELAVL4* and *PABPC4L* transcripts were upregulated in HH514–16 cells but not in BJAB cells. This result was consistent with the observation that *ELAVL4* and *PABPC4L* transcripts were upregulated in EBV(+) but not EBV(-) Akata cells crosslinked by anti-IgG (Fig 4A). These experiments demonstrated that EBV lytic activation caused by overexpression of the ZEBRA protein was sufficient to induce *PABPC4L* and *ELAVL4* expression in EBV-infected cells; no exogenous chemical or biologic stimulus was required to activate transcription of these two genes that encode RNA binding proteins.

### ZEBRA and BGLF5 protein turn down the expression of cellular proteins including IL6 and the RNA-binding proteins ELAVL4 and PABPC4L

**Fig 6:**
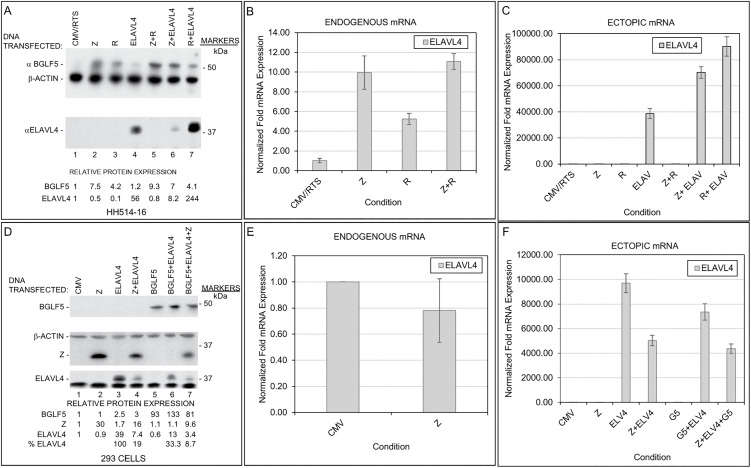
ZEBRA and BGLF5 turn down protein expression of the RNA-binding factor ELAVL4/HuD. HH514-16 **(A-C)** and 293 **(D-F)** cells were transfected with empty vectors CMV/RTS or CMV alongside vectors expressing *BZLF1* (Z), *BRLF1* (R), *BGLF5* (G5), *ELAVL4*, or combinations as indicated. Lysates were harvested after 24 hours and subjected to immunoblotting using indicated antibodies (**A, D**) or RT-qPCR analyses measuring endogenous (**B, E**) and ectopic *ELAVL4* transcripts **(C, F)**. Data are representative of two biological replicates; error bars represent SD from three technical replicates.

In earlier work, we had shown that ZEBRA and the BGLF5 proteins contribute to host shutoff by inhibiting protein translation [4]. However, the cellular targets of such protein synthesis shut off remain unknown. Since certain cellular transcripts were upregulated in lytic cells (Table 1, Figs 1–4), we asked if such selective increases were intended to ramp up the abundance of transcripts expressed at low levels or to counter host shutoff. To address this question, we introduced the *BZLF1*, *BRLF1* (encoding the other EBV lytic switch protein RTA), or *ELAVL4* plasmid alone or in different combinations into HH514–16 cells (Fig 6). Analysis of cell lysates 24 hours later showed that the BGLF5 protein was expressed, providing evidence of lytic cycle activation by *BZLF1* and *BRLF1* but not by the empty vector control (CMV/RTS; Fig 6A). As expected, introduction of *ELAVL4* resulted in expression of ELAVL4 RNA and protein (Fig 6A and 6C). However, co-expression of *BZLF1* inhibited the expression of ELAVL4 protein by ~85% (Fig 6A, lane 6 versus 4) despite ~2-fold (or ~100%) increase in the expression of *ELAVL4* transcripts when *BZLF1* plus *ELAVL4* were transfected compared to when *ELAVL4* was transfected alone (Fig 6C). By comparison, *BRLF1* did not blunt the expression of ELAVL4 protein. This result supported the idea that ZEBRA suppresses endogenous ELAVL4 protein expression – as indicated by *BZLF1*-mediated suppression of the endogenous ELAVL4 protein in lane 2 of Fig 6A despite a nearly 10-fold induction of the endogenous *ELAVL4* transcripts by *BZLF1* in Fig 6B. That said, it was difficult to distinguish whether BGLF5 protein also contributed to the suppression of ELAVL4 protein since, as expected, BGLF5 protein was expressed in cells induced into the lytic phase by *BZLF1* introduction. We therefore performed a similar experiment in 293 cells wherein introduction of *BZLF1* would not induce the expression of BGLF5 protein.

For the experimental results depicted in Fig 6D–6F, 293 cells were transfected with empty vector (CMV), *BZLF1*, *ELAVL4*, *BGLF5*, or indicated combinations of plasmids. As expected, introduction of *BZLF1* resulted in expression of ZEBRA but not BGLF5 protein after 24 hours (Fig 6D). Also, as expected, *BZLF1* did not induce ELAVL4 transcripts or protein (Fig 6D and 6E) – consistent with ZEBRA’s inability to induce *ELAVL4* transcription in EBV(-) Akata cells or BJAB cells (Figs 4A and S4). *BGLF5* also did not induce *ELAVL4* transcripts or protein (Fig 6D and 6F). Importantly, however, although the presence of *BZLF1* reduced the expression of the *ELAVL4* plasmid by <50% (possibly due to the presence of more than one plasmid), the abundance of ELAVL4 protein was reduced by 81% (Fig 6F, Fig 6D lanes 3 versus 4). In comparison, co-transfection of *BGLF5* resulted in ~20% reduction of *ELAVL4* RNA but ~67% drop in the abundance of ELAVL4 protein (Fig 6F, Fig 6D lanes 3 versus 6). Lastly, the effect of adding both *BZLF1* and *BGLF5* plasmids to *ELAVL4* plasmid was additive as ELAVL4 protein was reduced by >91% while its RNA abundance was reduced by ~50% (Fig 6F, Fig 6D lanes 3 versus 7). Thus, both ZEBRA and BGLF5 protein contributed to ELAVL4 protein shut off.

**Fig 7:**
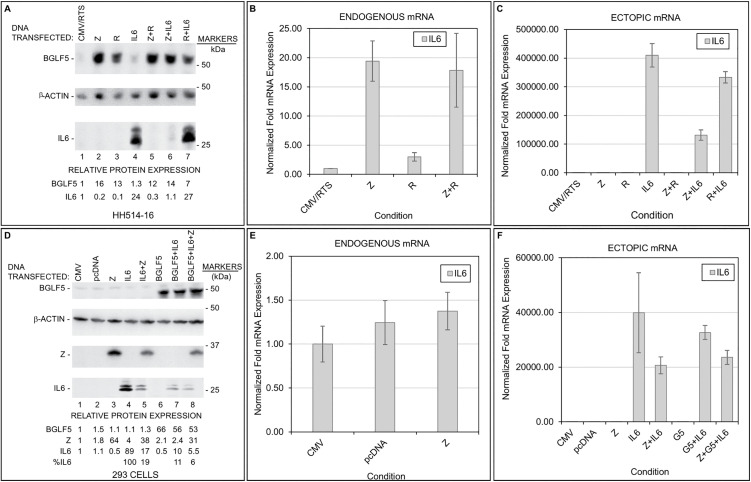
ZEBRA and BGLF5 turn down protein expression of cellular IL6 protein. HH514-16 **(A-C)** or 293 **(D-F)** cells were transfected with empty vectors CMV/RTS, CMV, or pcDNA alongside vectors expressing *BZLF1* (Z), *BRLF1* (R), *BGLF5*, *IL6*, or combinations as indicated. Lysates were harvested after 24 hours and subjected to immunoblotting using indicated antibodies (**A, D**) or RT-qPCR analyses measuring endogenous (**B, E**) and ectopic *IL6* transcripts **(C, F)**. Data are representative of two biological replicates; error bars represent SD from three technical replicates.

Mirroring the experimental set up in Fig 6, the experiments in Fig 7 examined the effects of *BZLF1*, *BRLF1*, and *BGLF5* on the transcription and protein expression of *IL6* whose transcripts we found were upregulated in lytic cells (Table 1, Figs 2 and 3). As shown in Fig 7A, *BZLF1* and *BRLF1* plasmids induced the expression of BGLF5 protein in HH514–16 cells. Like ELAVL4, endogenous IL6 protein was not observed after lytic activation by *BZLF1*, *BRLF1*, or both plasmids despite 15–20-fold induction of endogenous *IL6* transcripts by *BZLF1* and *BZLF1* + *BRLF1* (Fig 7A and 7B). Furthermore, while *BZLF1* suppressed *IL6* transcripts by 60% when co-transfected with *IL6* plasmid compared to when *IL6* plasmid was transfected alone, the suppressive effect of *BZLF1* on the expression of ectopic IL6 protein was more dramatic, i.e., 95% (Fig 7A and 7C). Again, *BRLF1* did not influence the expression of IL6. We note that transfection of *BZLF1* and *BRLF1* plasmids resulted in abundant expression of ZEBRA and RTA. Moreover, even though ZEBRA caused near total suppression of ELAVL4 and IL6 (Figs 6A and 7A), ELAVL4 and IL6 only modestly suppressed ZEBRA and RTA (S5 Fig).

In 293 cells, *BZLF1* transfection resulted in expression of ZEBRA but not BGLF5 protein, endogenous IL6 protein, or *IL6* RNA (Fig 7D and 7E). Like ZEBRA, ectopic expression of BGLF5 did not induce endogenous IL6 protein (Fig 7D). As for ectopic expression of IL6 by an *IL6* plasmid, while *BZLF1*, *BGLF5*, and *BZLF1* + *BGLF5* suppressed *IL6* transcripts by ~50%, ~ 15%, and ~40%, respectively, the suppressive effects on IL6 protein were more dramatic at 80%, 89%, and 94%, respectively (Fig 7D and 7F). These observations again point towards suppression of IL6 protein by ZEBRA and BGLF5 protein.

**Fig 8:**
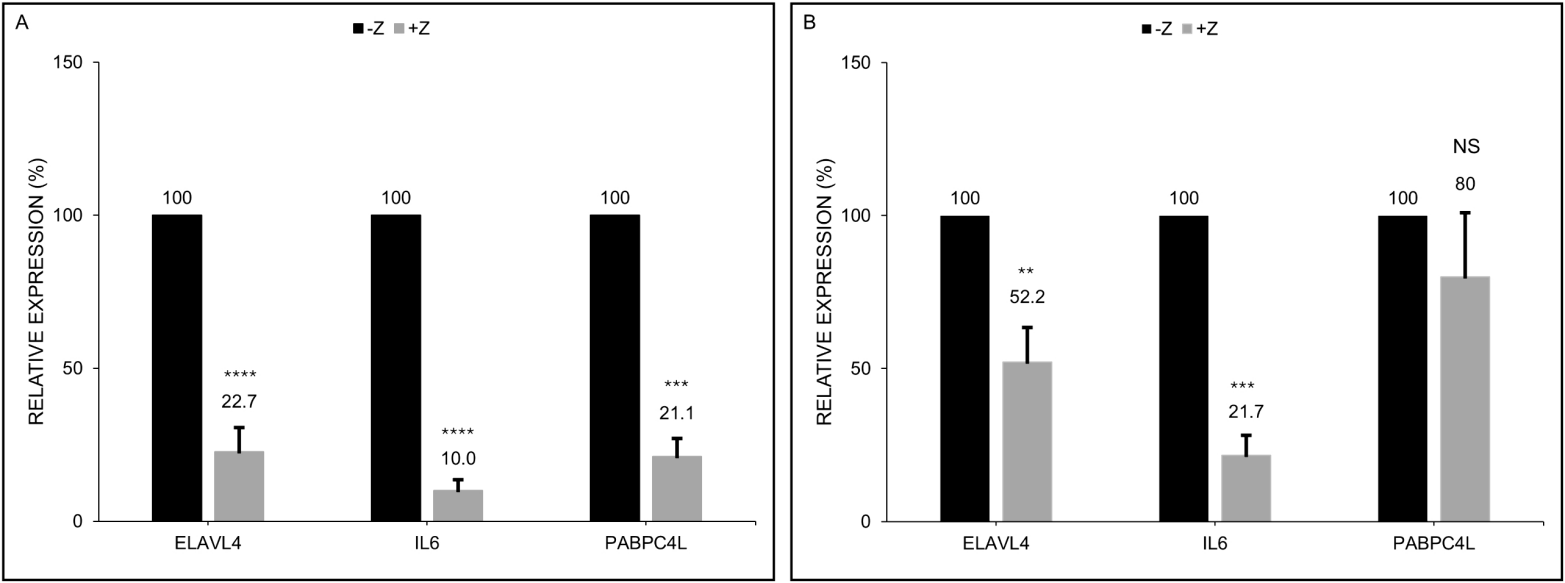
ZEBRA turns down the expression of cellular proteins ELAVL4, PABPC4L, and IL6. Twenty-four hours after transfection of plasmids encoding each of the three proteins ELAVL4, PABPC4L, or IL6 alone (-Z) or in combination with a *BZLF1* plasmid (+Z), lysates from HH514-16 cells (**A**) and 293 cells (**B**) were subjected to immunoblotting with antibodies targeting the cellular proteins. The graph represents aggregate data from 3 to 6 biological replicates; error bars represent SEM; NS, not significant; **, *p* < 0.01; ***, *p* < 0.001; ****, *p* < 0.0001.

Fig 8 summarizes aggregate results of the effects of ectopically expressed ZEBRA on the expression of ELAVL4, PABPC4L, and IL6 proteins following introduction of their respective plasmids in HH514–16 (Fig 8A) and 293 (Fig 8B) cells. We found that ZEBRA-induced lytic activation resulted in 80–90% suppression of the cellular proteins ELAVL4, PABPC4L, and IL6 when expressed from plasmids in HH514–16 cells (Fig 8A); in comparison, ZEBRA’s effects on the same proteins were less prominent in 293 cells (Fig 8B).

**Fig 9:**
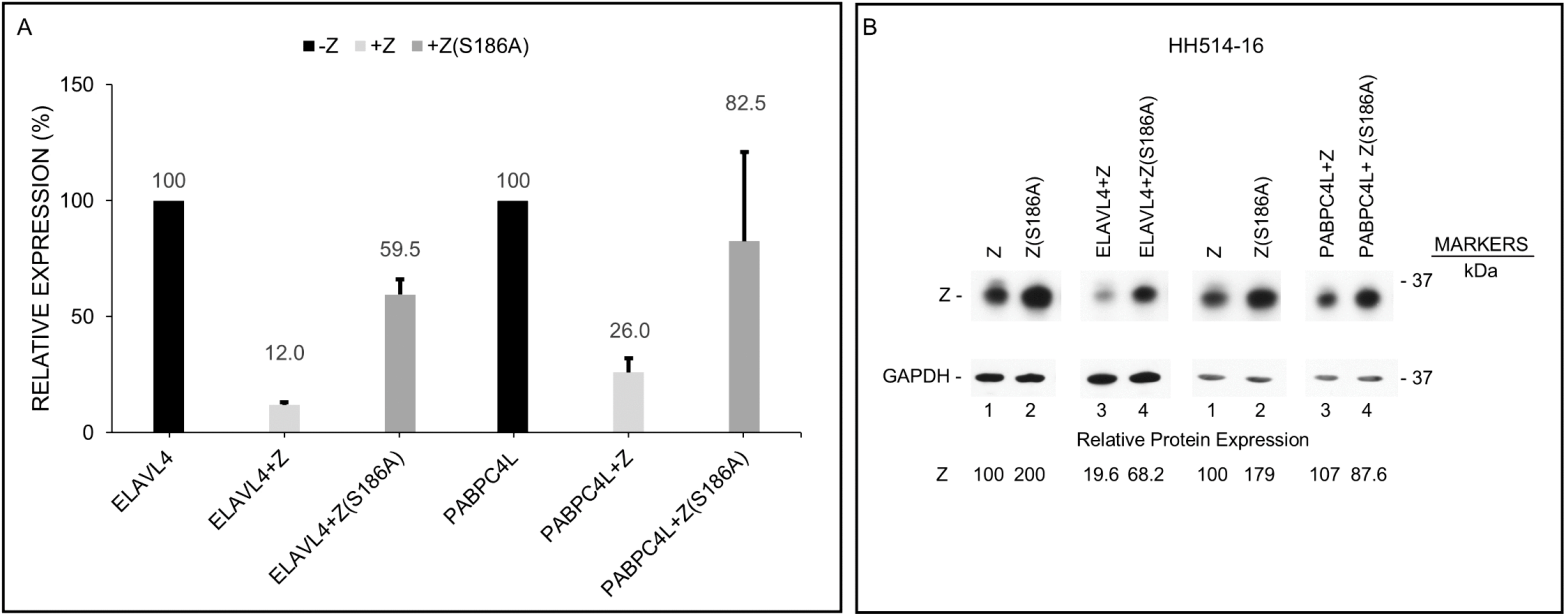
The S186A ZEBRA mutant is not as effective as wild-type ZEBRA at suppressing the expression of ELAVL4 and PABPC4L proteins. **(A)** HH514-16 cells were transfected with plasmids encoding *ELAVL4* or *PABPC4L* alone (-Z), or in combination with wild-type *BZLF1* (+Z) or S186A mutant *BZLF1* plasmid. Lysates were subjected to immunoblotting 24 hours later; graph shows aggregate data from two biological replicates. **(B)** Immunoblot of 24-hour lysates depicting the abundance of ZEBRA in the two biologic replicates.

Serine 186 is a critical residue in ZEBRA that enables transcriptional activation of EBV early lytic genes; mutation at this site impairs viral genome replication and blocks progression of the lytic cycle [26–30]. Given the importance of this serine residue also in nuclear translocation of PABPC (another poly-A binding protein) and ZEBRA-mediated translational shut off of proteins [4], we tested its contribution to ELAVL4 and PABPC4L protein shut off. Fig 9 shows that co-expression of wild-type ZEBRA suppressed the expression of ectopic ELAVL4 and PABPC4L in HH514–16 cells. However, although expressed well (Fig 9B), the S186A ZEBRA mutant was less effective at dampening the expression of ELAVL4 and PABPC4L proteins (Fig 9A). These results, demonstrating the importance of the S186 residue in suppressing protein levels of ELAVL4 and PABPC4L, are consistent with our prior observation that the S186 residue is important in shutting off protein expression [4]. A noteworthy observation in the collective context of these experiments, was that introduction of *BZLF1* increased the expression of both endogenous and ectopic *ELAVL4* and *IL6* transcripts in HH514–16 cells in which BGLF5 protein was well expressed. This result suggests that *ELAV4* and *IL6* RNA may be resistant to the BGLF5 nuclease activity, i.e., the canonical mode of host shut off (Figs 6A–6C and 7A–7C).

### ZEBRA uses protein shut off to tightly regulate the abundance of select viral proteins

**Fig 10:**
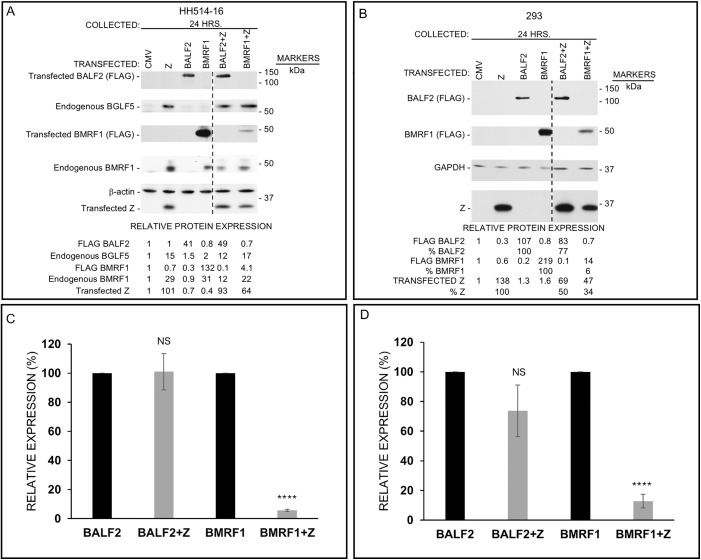
ZEBRA tightly regulates the expression of select viral proteins during the lytic phase. HH514-16 **(A, C)** and 293 **(B, D)** cells were transfected with an empty vector (CMV), *BZLF1* plasmid (Z), *BMRF1* plasmid, *BALF2* plasmid, or combinations as indicated. Lysates were harvested 24 hours later and subjected to immunoblotting using indicated antibodies. Ectopically expressed BALF2 and BMRF1 proteins were detected using anti-FLAG antibodies while ectopically expressed ZEBRA and endogenous BMRF1 and BGLF5 were detected using target-specific antibodies. Graphs representing aggregate data from 3 to 6 biological replicates are depicted in C and D; error bars represent SEM; NS, not significant; ****, *p* < 0.0001.

To address if ZEBRA’s ability to shut off expression of cellular proteins also extended to viral proteins, we selected two key lytic phase proteins encoded by *BMRF1* and *BALF2*. *BMRF1* encodes EA-D, the DNA polymerase processivity factor, and *BALF2* encodes the single-stranded DNA binding protein. Both proteins are critical for replication of the viral genome during the lytic phase. In addition, EA-D ensures a smooth transition from expression of early lytic genes to replication of the episomal genome early during the lytic phase [31]. In an experiment that was similar in design to those in Figs 6 and 7, introduction of *BZLF1* into HH514–16 cells for 24 hours resulted in expression of ZEBRA, endogenous BGLF5 protein, and endogenous EA-D (Fig 10A). This result was expected as *BGLF5* and *BMRF1* are transcriptional targets of ZEBRA. Introduction of FLAG-tagged *BALF2* and *BMRF1* resulted in the expression of tagged proteins. When *BZLF1* and *BALF2* were co-transfected, there was no effect on the abundance of ectopically expressed BALF2 protein (lanes 3 versus 6); as expected, endogenous BGLF5 protein was also expressed. However, when *BZLF1* was introduced alongside ectopic *BMRF1*, the abundance of FLAG-EA-D was dramatically reduced, i.e., by 97% compared to *BMRF1* transfection alone (lanes 4 versus 7); of note, the reduction in endogenous EA-D was only 29% and BGLF5 protein was abundantly expressed. To assess the contribution of ZEBRA versus BGLF5 protein to the suppression of ectopic EA-D, we again utilized 293 cells. As shown in Fig 10B, the presence of ZEBRA minimally affected the expression of ectopic BALF2 protein (lanes 3 versus 5). However, co-expression of ZEBRA resulted in 94% suppression of EA-D (lanes 4 versus 6), thereby implicating ZEBRA in selective shut off of viral proteins to maintain “optimal” levels of such proteins during the lytic phase.

Collectively, our results indicate that the EBV lytic switch protein ZEBRA shuts off the expression of cellular proteins such as the RNA binding factors ELAVL4 and PABPC4L while simultaneously activating their transcription – countering host shut off. Beyond cellular proteins, ZEBRA also tightly regulates the abundance of key viral proteins during the lytic phase.

### ELAVL4 and PABPC4L, upregulated in lytic cells by ZEBRA, regulate EBV lytic transcript abundance, viral genome replication, and virus release

**Fig 11:**
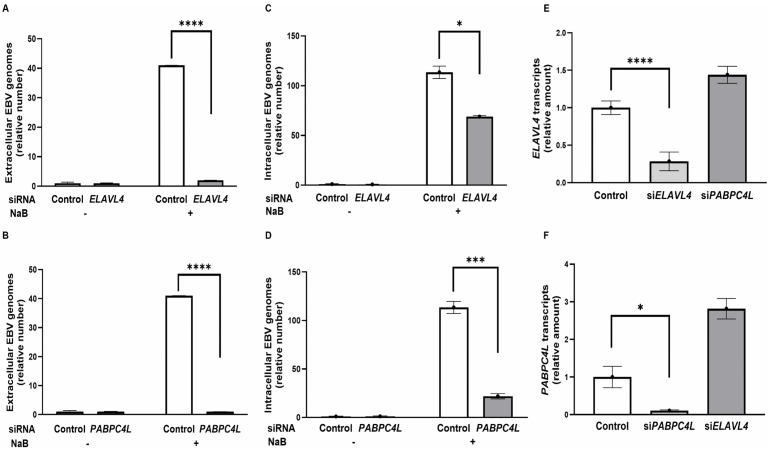
Depletion of *ELAVL4* or *PABPC4L* transcripts inhibits EBV release and lytic genome replication. HH514-16 BL cells were transfected with control/non-targeting siRNA (white bars) versus siRNAs targeting *ELAVL4* (light gray bars) or *PABPC4L* (dark gray bars). After 24 hours, cells were treated with NaB to induce the lytic phase and harvested at different time points: At 48 hours post-induction, DNase-treated culture supernatants **(A and B)** or cell extracts **(C and D**) were analyzed by qPCR using primers targeting *BALF5* to quantify encapsidated virus (A and B) or intracellular genomes **(C and D)**. At 24 hours post-induction, *ELAVL4* and *PABPC4L* transcripts were quantified using RT-qPCR **(E and F)**. Data in A-D were normalized to untreated, control siRNA-transfected cells; data in E-F were normalized to control siRNA-transfected cells. Error bars, SEM of 3 biological replicates, with 3 technical replicates within each biological replicate; *, p < 0.05; ***, p < 0.001; ****, p < 0.0001.

The paradoxical regulation by ZEBRA – upregulating *ELAVL4* and *PABPC4L* transcription while simultaneously suppressing their protein abundance – prompted us to investigate whether ELAVL4 and PABPC4L functionally contribute to progression of the lytic cycle. We therefore depleted ELAVL4 and PABPC4L using siRNAs and quantified the amounts of encapsidated virus using qPCR of the EBV *BALF5* (DNA polymerase) gene in DNase-treated culture supernatants. As shown in Fig 11A and 11B, induction of the lytic cycle in HH514–16 cells using NaB for 48 hours resulted in ~40-fold increase in the release of encapsidated EBV genomes. By comparison, virus release was almost completely abrogated when ELAVL4 or PABPC4L was depleted. We next asked if the loss of release of packaged EBV genomes from cells was due to a defect in replicating the viral genomes – and therefore assayed intracellular EBV genome amplification in HH514–16 cells using qPCR targeting *BALF5*. Twenty-four hours after NaB exposure, there was > 100-fold increase in the number of EBV genomes; however, compared to control siRNA, there was ~ 40% reduction in the number of intracellular genomes following siRNA-mediated depletion of ELAVL4; the effect was even greater, i.e., ~ 80% reduction in EBV genome amplification when PABPC4L was depleted (Fig 11C and 11D). Using shRNAs that target distinct regions of *ELAVL4* and *PABPC4L*, we confirmed that knock down of these genes suppressed viral genome replication in both HH514–16 cells and an LCL (S6A and S6B Fig). As expected, siRNAs resulted in depletion of transcripts from their cognate targets (Fig 11E and 11F); similarly, shRNAs resulted in knockdown of *ELAVL4* and *PABPC4L* transcripts and HuD protein (S6C and S6D Fig). Thus, ELAVL4 and PABPC4L contributed to viral genome replication, and ultimately, release of encapsidated virus.

**Fig 12:**
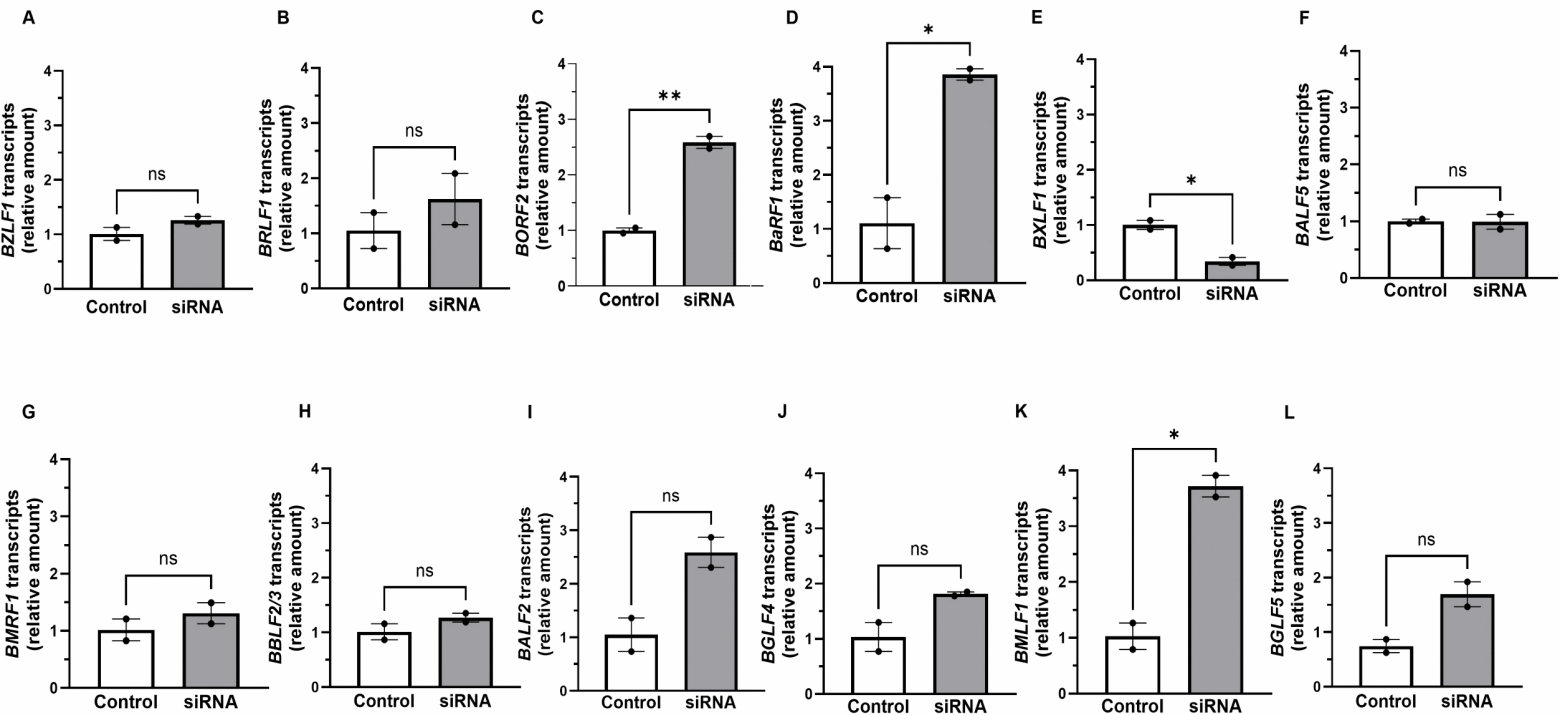
Knockdown of ELAVL4 reduces the abundance of *BXLF1* transcripts while increasing the abundance of *BORF2*, *BaRF1*, and *BMLF1* transcripts. HH514-16 BL cells transfected with control/non-targeting RNA (white bars) or siELAVL4 (gray bars) were treated with NaB to induce the lytic phase (as part of the experiment presented in Fig 11). Cells were harvested after 24 hours and subjected to RT-qPCR using primers targeting: **(A)** *BZLF1*, **(B)** *BRLF1*, **(C)** *BORF2*, **(D)** *BaRF1*, **(E)** *BXLF1*, **(F)** *BALF5*, **(G)** *BMRF1*, **(H)** *BBLF2/3*, **(I)** *BALF2*, **(J)** *BGLF4*, **(K)** *BMLF1*, and **(L)** *BGLF5*. Data were normalized to control siRNA-transfected cells. Error bars, SEM of 3 biological replicates, with 3 technical replicates within each biological replicate; ns, not significant; *, p < 0.05; **, p < 0.01.

**Fig 13:**
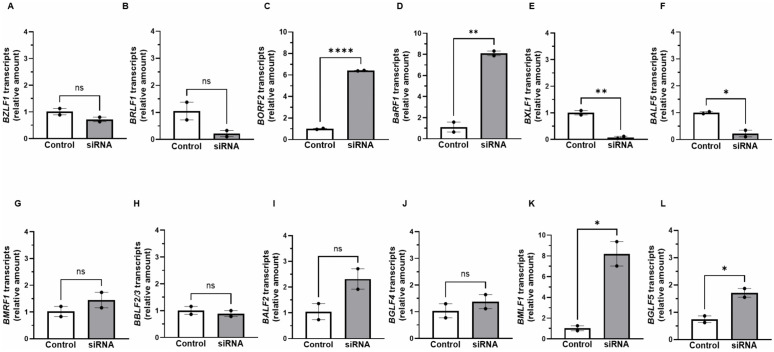
Knockdown of PABPC4L reduces the abundance of *BXLF1* and *BALF5* transcripts but increases the abundance of *BORF2*, *BaRF1*, and *BMLF1* transcripts. HH514-16 BL cells transfected with control RNA (white bars) or siPABPC4L (gray bars) were treated with NaB to induce the lytic phase (as part of the experiment presented in Fig 11). Cells were harvested after 24 hours and subjected to RT-qPCR using primers targeting: **(A)** *BZLF1*, **(B)** *BRLF1*, **(C)** *BORF2*, **(D)** *BaRF1*, **(E)** *BXLF1*, **(F)** *BALF5*, **(G)** *BMRF1*, **(H)** *BBLF2/3*, **(I)** *BALF2*, **(J)** *BGLF4*, **(K)** *BMLF1*, and **(L)** *BGLF5*. Data were normalized to control siRNA-transfected cells. Error bars, SEM of 3 biological replicates, with 3 technical replicates within each biological replicate; ns, not significant; *, p < 0.05; **, p < 0.01; ****, p < 0.0001.

**Fig 14:**
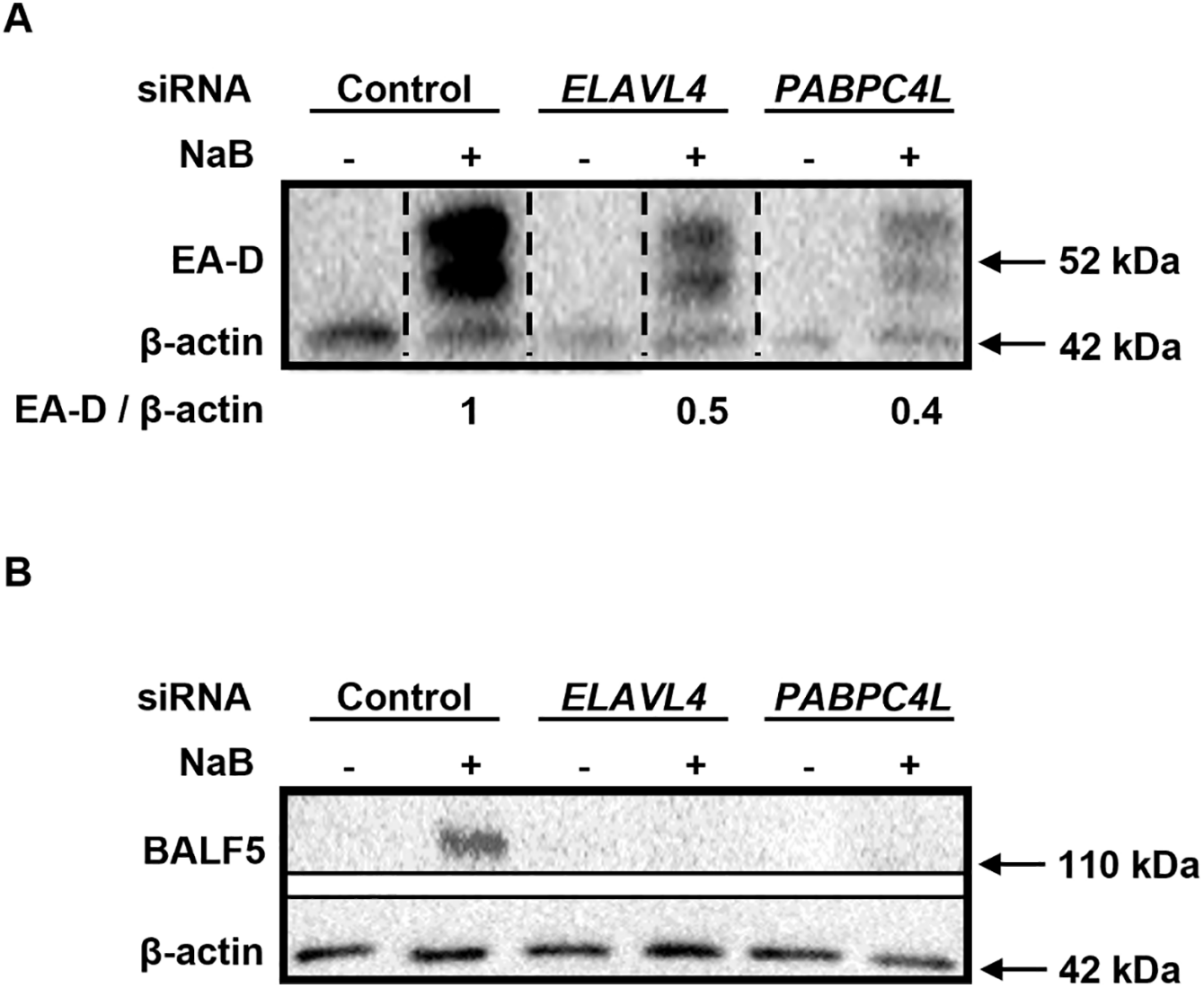
Depletion of ELAVL4 or PABPC4L reduces the abundance of viral DNA replication proteins EA-D and BALF5. EBV^+^ HH514-16 BL cells were exposed to control siRNA versus siRNAs targeting *ELAVL4* or *PABPC4L* followed by treatment with NaB 24 hours later. Cells lysates were processed after another 24 hours for immunoblotting with antibodies targeting the viral DNA polymerase processivity factor (EA-D; **A**) and DNA polymerase (BALF5; **B**).

EBV early lytic genes encode several key components of the DNA replication machinery and are therefore necessary for viral genome replication. To assess if the effects of the RNA binding factors ELAVL4 and PABPC4L on viral genome replication were related to the abundance of EBV early lytic transcripts, we used RT-qPCR to measure the steady state levels of transcripts from a dozen EBV early lytic genes after depletion of ELAVL4 or PABPC4L. Fig 12E shows that compared to control siRNA, depletion of *ELAVL4* resulted in significant reduction of *BXLF1* transcripts in NaB-exposed HH514–16 cells. In comparison, while depletion of *PABPC4L* similarly resulted in a reduction in *BXLF1* transcripts, it also reduced the abundance of *BALF5* transcripts (Fig 13E and 13F). As noted above, *BALF5* encodes the viral DNA polymerase; *BXLF1* encodes the enzyme thymidine kinase. While depletion of *ELAVL4* or *PABPC4L* did not affect most of the other transcripts, *BORF2*, *BaRF1*, and *BMLF1* transcripts were elevated upon depletion of either *ELAVL4* or *PABPC4L* (Figs 12C, 12D, 12K, 13C, 13D and 13K). *BORF2* and *BaRF1* encode the large and small subunits of the viral ribonucleotide reductase, respectively; *BMLF1* encodes SM, an RNA binding protein that post-transcriptionally regulates viral and cellular transcripts. Using antibodies that reliably detect two essential viral replication proteins, the DNA polymerase BALF5 and the DNA polymerase processivity factor EA-D, by immunoblotting, we also found that depletion of *ELAVL4* or *PABPC4L* resulted in 50–60% reduction in EA-D protein (including the phosphorylated isoform; Fig 14A) and near-total loss of BALF5 (Fig 14B) in NaB-treated cells. Of note, the lack of effect of *ELAVL4* depletion on *BGLF5* transcripts (Fig 12L) was consistent with a lack of effect of ELAVL4 overexpression on BGLF5 protein in lytically induced HH514–16 cells in Fig 6A. These experiments support the conclusion that two RNA binding factors, that are subject to complex regulation during the lytic phase, in turn regulate the abundance of transcripts from select early lytic genes while also supporting the expression of key DNA replication proteins to ensure EBV genome replication, and ultimately, release of the virus.

## Discussion

Lytic reactivation of EBV from latency is essential for propagation and maintenance of the virus within the human population. The lytic cycle is also a critical component of the pathogenesis of virus-associated diseases [32–37]. While large DNA viruses like EBV encode many RNAs and proteins necessary for their replication and persistence within host cells, many cellular gene products must also play an essential role in the viral life cycle. However, the contribution of cellular gene expression during the lytic phase of the EBV life cycle remains underexplored. Investigation of cellular events that occur at early times prior to reactivation may help elucidate the mechanism(s) regulating the switch between latency and the lytic cycle [38]. It is equally important to understand how the viral lytic cycle itself alters expression of cellular genes to ensure progress of the lytic phase. In this report, we concentrate on cellular gene expression that is specific to cells that enter the EBV lytic cycle; products of such genes are needed to overcome host shut off and ensure completion of the viral lytic cycle.

Our previous studies led to the concept that subpopulations of cells, either refractory or susceptible to EBV lytic reactivation exhibited different patterns of cellular gene expression [14,17,18,39]. Here, we identify additional cellular genes that are upregulated in the refractory cells but also identify the transcripts of several cellular genes that accumulate specifically in the lytic subpopulation of HH514–16 cells regardless of the inducing stimulus. Expression of these cellular genes is blocked by VPA that inhibits EBV lytic reactivation. These transcripts are also upregulated in EBV(+) but not in EBV(-) Akata cells treated with anti-IgG. The cellular transcripts are kinetically downstream of viral early lytic genes. Expression of a subset of cellular transcripts that encode two RNA binding proteins, ELAVL4 and PABPC4L, is triggered by introduction of a plasmid expressing the EBV lytic cycle activator ZEBRA only into EBV-infected cells – and help overcome ZEBRA and BGLF5-induced shut off of ELAVL4 and PABPC4L proteins. This escape strategy is important as we find that ELAVL4 and PABPC4L regulate the abundance of early lytic gene products that are essential for viral genome replication.

Host cell shut off of alpha- and gammaherpesviruses presents a dilemma: how are transcripts of viral genes, and of some cellular genes, protected from the shut off and in some instances upregulated? In Herpes simplex virus (HSV) infection, the virion host shutoff protein vhs, degrades both cellular and viral mRNA; the high transcription rate of the viral genes is thought to overwhelm this mechanism [40]. As for cellular genes, some are activated and their transcripts stabilized at the same time that the vast majority of cellular gene expression is repressed [41,42]. The finding that vhs selectively degrades some mRNAs suggests that some intrinsic properties of transcripts, either specific sequences or structural motifs, may protect them from the host shut off [40]. Indeed, the 3’ untranslated regions of *IL6*, *GADD45B*, and *C19ORF66* transcripts in KSHV-infected cells demonstrate the presence of SOX-resistance elements whose interactions with host ribonucleoprotein complexes mediate escape from host shut off [43–46]. Other cellular transcripts in KSHV-infected cells escape host shut off by the viral ORF50 protein-mediated transcriptional upregulation, demonstrated to occur even independently of the presence of the KSHV genome [47]. In an earlier study of nascent transcriptomes, we identified a set of upregulated cellular transcripts that contributed to EBV lytic reactivation and lytic cycle progress; however, whether these transcripts were upregulated by viral or cellular factors and whether such upregulation was required to overcome host shut off is unclear [5]. In the present study, we identified several cellular targets that are transcriptionally upregulated in lytic cells to counter host shut off that is also partially mediated by ZEBRA. We have previously shown that ZEBRA is a viral host shutoff factor that efficiently inhibits global protein synthesis in the absence of any other viral proteins [4].

Beyond turning the lytic phase on, ZEBRA also tightly regulates the abundance of viral gene products. While the single-stranded DNA binding protein (*BALF2* gene product) and EA-D (*BMRF1* gene product) are both essential for viral genome replication during the lytic phase, ZEBRA shuts off the ectopic expression of EA-D protein while permitting ectopic expression of the BALF2 protein. Although the reasons for such nuanced regulation are unclear, we speculate that it may be because EA-D performs several functions during the lytic cycle. Aside from serving as a clamp for the viral DNA polymerase to ensure processivity of genome replication, EA-D functions as a transcriptional coactivator, and importantly also, ensures a smooth transition from early lytic gene transcription to replication of the viral episome [31,48]. Untimely and excessive expression of EA-D may lead to deregulation of the kinetically coordinated events that ensure the completion of the lytic cycle. A second mechanism by which ZEBRA influences lytic gene expression is through induction of cellular RNA-binding proteins such as ELAVL4 and PABPC4L that modulate the abundance of early lytic transcripts and proteins. Depletion of these RNA-binding factors reduces *BALF5* (viral DNA polymerase) transcripts and protein, *BXLF1* (viral thymidine kinase) transcripts, and EA-D protein, consistent with impaired viral DNA replication. In contrast, an explanation for the concurrent increase in *BORF2*, *BaRF1*, and *BMLF1* transcripts is less straightforward. Given their roles in maintaining the ribonucleotide pool (BORF2 and BaRF1, subunits of ribonucleotide reductase) and regulating post-transcriptional processes (*BMLF1*/SM protein), it is likely that ELAVL4 and PABPC4L fine-tune both nucleotide availability and RNA metabolism to support the transcriptional demands of the lytic cycle.

ZEBRA has been shown to be a potent activator of host shutoff in both EBV(+) and EBV(-) cell lines [4]. ZEBRA-induced host shutoff involves three mechanisms: global shut down of the initiation of protein synthesis, blockade of nuclear export of RNA, and nuclear translocation of cytoplasmic PABPC. Point mutations of ZEBRA that caused diminished PABPC nuclear translocation led to reduced inhibition of translation. Importantly, ZEBRA’s ability to induce these three activities in EBV(-) cells indicated that ZEBRA directly induces cellular host shutoff in the absence of any other viral factors, including the nuclease encoded by *BGLF5* [4]. Our present work indicates that serine 186, a residue that is critical for ZEBRA’s recognition of methylated promoters and therefore transcriptional activation of early lytic genes, also contributes to its ability to suppress protein expression. ZEBRA-mediated transcriptional upregulation of endogenous *ELAVL4* and *PABPC4L* transcripts to counter protein suppression only occurs in EBV-infected cells (but not in EBV(-) Akata cells, BJAB cells, or 293 cells), indicating that other viral factors likely regulate the specific increase in these transcripts; however, intriguingly, the abundance of *PABPC4L* transcripts appears to also depend on viral genome replication in LCLs (S3 Fig).

To distinguish transcript accumulation resulting from EBV lytic induction versus from pleiotropic effects of exogenous stimuli, we examined cellular gene expression following ZEBRA transfection. This revealed that genes encoding mRNA-binding proteins – ELAV-like and PABP-like – are specifically upregulated during the EBV lytic phase. Notably, of the *ELAVL* genes, *ELAVL3* and *ELAVL4*, but not *ELAVL1* or *ELAVL2*, were induced. While ELAVL1 is broadly expressed, ELAVL2–4 are largely neuronal and critical for differentiation and development [49–52]. ELAV proteins stabilize AU-rich mRNAs and can either repress or enhance translation depending on context [53–59]; for example, ELAVL4 interacts with eIF4A to promote cap- and poly(A)-dependent translation [60] – supporting our observation that depletion of ELAVL4 suppresses EA-D and BALF5 proteins even without affecting their respective transcripts. ELAVL4/HuD is also known to stabilize *CDKN1A* transcripts that encode p21, a CDK inhibitor that blocks G1-S transition, a key lytic phase observation attributed to ZEBRA [61,62]. Although ELAVL3 and ELAVL4 have not previously been linked to viral infection, related family members modulate diverse viruses: ELAVL1 regulates HPV late gene expression, supports HCV replication, stabilizes Sindbis virus RNAs, and is relocalized by KSHV kaposin-B to enhance mRNA stability [63–67]. Our findings show that ELAVL3 and ELAVL4, normally neuron-restricted, are robustly induced in EBV-lytic Burkitt lymphoma cells, where basal expression is otherwise minimal.

We found that *ELAVL4* and *PABPC4L* are induced during the later stages of the EBV lytic cycle (12–24 hours post-reactivation), consistent with their observed roles in regulating transcripts and proteins encoded by early lytic genes. PABPC4L is a poorly characterized homolog of PABPC4 (inducible PABP, iPABP), which itself shares ~79% amino acid identity with the major cytoplasmic poly(A)-binding protein PABPC1 and is rapidly induced upon T-cell activation. Like PABPC1, iPABP contains four conserved RNA-recognition motifs (RRMs), an unstructured linker, and a C-terminal protein interaction domain. In contrast, PABPC4L encodes a predicted 370-aa protein containing the four RRMs but lacking the linker and C-terminal regions, with ~75% identity to iPABP across the RRMs [68,69]. The specific induction of PABPC4L during lytic EBV replication raises the possibility that it compensates for the loss of cytoplasmic PABPC1, which undergoes nuclear translocation during lytic reactivation of EBV and KSHV [4,70]. Analogous viral strategies support this idea: rotavirus NSP3 displaces PABP from eIF4G yet substitutes for its function to promote viral mRNA translation, while poliovirus protease 2A cleaves PABP, rendering host translation dependent on IRES-driven mechanisms [71–74]. Since EBV and KSHV transcripts resemble cellular mRNAs (5′ capped, polyadenylated, and spliced), they still require canonical translation machinery. Thus, induction of PABPC4L may preserve viral mRNA translation under conditions where PABPC1 is sequestered. Notably also, iPABP is thought to stabilize and promote translation of cytokine mRNAs during T-cell activation [69] – a situation reminiscent of the transcriptional surge during lytic EBV reactivation. By analogy, PABPC4L could support translation of viral and select host transcripts during lytic infection.

Our approach, using sorted lytic or refractory cells rather than bulk populations, allowed us to ask two questions: 1) Do refractory cells respond to the inducing stimulus? and 2) Does cellular gene expression in response to the inducing stimulus differ between those cells that enter the lytic cycle and those that remain refractory to lytic induction? Our initial analyses revealed that cells refractory to lytic induction do respond to the inducing stimulus. For example, when HH514–16 cells were treated with NaB and then sorted, both refractory and lytic subpopulations exhibited an increase in global acetylation on the tail of histone H3 [14]. This result indicated that refractory cells were responsive to the HDAC inhibitory effects of NaB. Moreover, in response to the HDAC inhibitors NaB or TSA, expression of some cellular genes, including *STAT3*, *FOS* and *IL8*, and those encoding components of the heterochromatin silencing machinery including KRAB-ZFPs and the histone methyltransferase SETDB1 were specifically increased in refractory cells [14,17]. In lytic cells, the transcript levels of most of the cellular genes we investigated were similar to or lower than untreated controls. However, several genes, including *IL6*, were preferentially upregulated in the lytic subpopulation [14,17]. Our present work indicates that this upregulation of IL6 mRNA mitigates ZEBRA-mediated shut off of IL6 protein. That IL6 expression is up-regulated in KSHV [75] and EBV infection suggests that the behavior of some cellular transcripts during viral lytic replication is conserved through evolution.

EBV infection and the IL6 pathway are closely linked. EBV infection induces B cells to express IL6 and IL6R [76,77]. Binding of the virion glycoproteins gp350 and gp220 to the cellular receptor CD21 leads to increases in IL6 transcript and protein levels [76]. Expression of the EBV latent membrane protein LMP1 also induces IL6 production [78]. Products of the early EBV lytic genes, *BZLF1* and *BRLF1* activate IL6 production in early-passage LCL [32]. The combination of ZEBRA and LMP1 increases IL6 levels significantly more than either protein alone [32]. Although LMP1 is not expressed in HH514–16 cells during latency, the LMP1 transcript and protein are highly expressed specifically in the lytic population [14]. LMP1 in lytic HH514–16 cells may cooperatively activate IL6 together with other EBV lytic proteins. IL6 is likely to play many roles in the viral life cycle and in EBV-associated diseases. IL6 can act via autocrine or paracrine mechanisms as a growth factor for EBV-infected lymphocytes [79,80]. High levels of IL6 inhibit the activity of natural killer cells that are a first-line innate defense that limits EBV-induced B cell transformation until adaptive virus-specific immune mechanisms develop [81,82]. Induction of IL6 during lytic infection could therefore enhance the transformation or growth of newly infected cells and mediate escape from innate immunity of the host [82]. Indeed, IL6 is thought to play a crucial role in EBV-induced lymphoproliferative diseases (LPD), and, neutralizing monoclonal antibodies to human IL6 enhanced the survival of SCID mice injected with human peripheral blood leukocytes isolated from EBV-positive donors [83]. In addition, EBV early lytic viral proteins enhance tumor formation in SCID mice, likely by enhancing IL6 secretion [32]. A clinical trial showed that treatment with a monoclonal anti-IL6 neutralizing antibody often resulted in remission of LPD [84]. Elevated levels of EBV DNA are often a predictive marker of LPD [85–87], and it has been suggested that lytic viral reactivation after organ transplant correlates strongly with the risk of LPD [88]. These studies suggest that activation of IL6 during the EBV lytic phase plays an important role in EBV-induced lymphoproliferation in cell culture, in experimental animals, and in humans.

## Materials and methods

### Cell lines

The EBV-infected HH514–16 Burkitt lymphoma cell line is a sub-clone of the P3J-HR1K Burkitt lymphoma cell line [16]. The EBV(+) and EBV(-) Akata Burkitt lymphoma cell lines were generously supplied by Kenzo Takada [11,89]. The LCL was previously published [90]. The B-cell lymphoma line, BJAB, served as an EBV-negative control as did 293 cells [91]. Cells were cultured in RPMI-1640 containing 8% or 10% fetal bovine serum, penicillin (50 U/ml), streptomycin (50 U/ml), and amphotericin B (1 µg/ml). Cells were grown at 37°C under 5% CO_2_.

### Chemical treatment of cell lines

Cells were sub-cultured at 3–5 x 10^5^/ml and 24–48 hours later treated with chemical stimuli. NaB (Sigma no. B5887) was used at 3 mM, TSA (Sigma #T8552; WAKO Chemicals USA #209–17563) was used at 5 μM, AzaCdR (Sigma #A3656) was used at 5 μM, VPA (Sigma #P4543) was used at 10 mM, TPA (Sigma #524400) was used at 20 ng/ml, and phosphonoacetic acid/PAA (Sigma #284270) was used at 300 µg/ml. Rabbit anti-human IgG (anti-IgG) (Dako #A042301-2) was used at 7.5 µg/ml.

### Cell sorting

Separation of lytic and refractory cell populations was performed as described previously using reference EBV-positive and EBV-negative human sera [13].

### Transfections

HH514–16 cells were sub-cultured to 3–5 x 10^5^ cells/ml and transfected 24–48 hours later when cell counts were approximately 1 x 10^6^/ml. 10 μg of plasmid DNA were mixed with 1.5x10^6^ cells in 400 μl of RPMI1640 plus 8% fetal bovine serum. The cells were exposed to 0.25 kV in a BioRad gene pulse unit. In the experiments shown in Figs 11–14 and S3, plasmid DNA (10–20 μg) or siRNA (200 pmol) were transfected into 1 x 10^6^ cells using Ingenio solution (MIR50117, Mirus BioSolutions) and an Amaxa Nucleofector II (program A-024) as previously described [92]. Electroporated cells were sub-cultured at 1 x 10^6^ cells/ml at 37°C in 5% CO2 and harvested at timepoints indicated in the respective figures. 293 cells were grown to 80% confluence at which time they were reseeded in a 6-well plate at 0.3 x 10^5^/well in 2 ml medium. In 2–3 days, when cells reached 50% confluence, they were transfected via Lipojet (SignaGen laboratories) or DMRIEC reagent (Life Technologies) and harvested at timepoints indicated in the figures.

### Lentivirus transduction

HEK-293T cells at 70–80% confluence were transfected with lentiviral packaging plasmids and shRNA constructs. After 18–20 hours, the media were replaced, and viral supernatant was collected 48 h later. The supernatant was clarified by centrifugation at 350 × g for 5 min and filtered through a 0.45 μm PES syringe filter. Lentivirus was concentrated using Lenti Concentrator (Origene, cat# TR30026). HH514–16 Burkitt lymphoma cells and LCLs were transduced with 100 μl of concentrated virus in the presence of 8 μg/ml Polybrene. Spinoculation was performed at 800 × g for 90 min at room temperature. Transduction was performed on two consecutive days. For stable cell line generation, cells were selected in puromycin-containing media for 10 days.

**Plasmids:** *FLAG*-*BZLF1* plasmid, used in S3 Fig, was described previously [31]. Other plasmids included CMV (pHD1013), CMV/FLAG/BALF2, CMV/FLAG/BMRF1, CMV/gZ [29], CMV/gZS186A [29], CMV/RTS [93], R(pRTS/RTA) [93], pcDNA3.1, pcDNA3.1/HuIL6, ELAVL4 (CMV/V6-XL5/ELAVL4 – Origene NM_021952#S0313227), and PABPC4L (pCMV6-ENTRY/PABPC4L/FLAG – Origene RC225562).

**siRNAs:** Sequences of siRNAs, ordered from Thermo Fisher, are shown below:

*ELAVL4* siRNA: 5’ GAAUAUGACCCAAGAAGAATT 3’
*PABPC4L* siRNA: 5’ GGUGAUGAGUGAUGAUCAATT 3’

**shRNA** sequences are shown below:

*ELAVL4*: 5’ AAGTCACGAATCACCTTTACG 3’ (Horizon Discovery, Oligo ID: TRC0000038707

provided in the pLKO.1 lentivirus plasmid)

*PABPC4L*: 5’ TCTCCTGAGGATGCTACTAAA 3’ (Vector Builder, VectorID: VB260204–1449uwy; vector name pLV[shRNA]-Puro-U6 > hPABPC4L[shRNA#3])

### Extraction of RNA and DNA

Total RNA was extracted from samples using Qiagen RNeasy/RNeasy Plus mini kits (#74106/74134) or Invitrogen Purelink RNA mini kit. Total genomic DNA was extracted using Invitrogen™ Purelink™ DNA mini kit. RNA/DNA concentration was determined using a NanoDrop (ThermoFisher).

### RT-qPCR

The relative levels of selected transcripts were measured using RT-qPCR with gene-specific primers using the iScript SYBR Green RT-PCR kit (Bio-Rad). Relative expression levels were calculated using the ΔΔCt method, normalized to 18S rRNA. Individual samples were assayed in triplicate. SABiosciences was the source of primers for ELAVL3 (cat # PPH07095A) and ELAVL4 (cat # PPH09946A). All other primers were designed using Primer3 [94] or have been described previously [14,95,96]. See Table 4 for sequences of primers not previously described.

**Table 4:** Sequences of primers used to detect expression of viral and cellular transcripts.

Gene Name	Forward Primer	Reverse Primer	Product (bp)
*IL6*	TAC CCC CAG GAG AAG ATT CC	GCC ATC TTT GGA AGG TTC AG	199
*IL6R*	AAG ACC CCC ACT CCT GGA ACT	CGT GGA TGA CAC AGT GAT GCT	126
*PABPC4L*	AAT GTG GCA GCT ATG GAA CC	AGA TGA AAG GCA GGA GAG CA	166
*CAMTA1*	AGT GCA GAA AAT GAA GAA TGC G	CAA AAT TCT CCT GCT TGA TTC G	115
*CAMK2B*	GCA AAA GCT ACA GTG GGA GC	CGC CTT ATC TCC ATA GCT GC	154
*JUN*	TCC CCT AAC CTC TTT TCT GC	AAC ATC GCA CTA TCC TTT GG	236
*RAB27B*	CGA ATG GAT CTT CAG GGA AA	CCA TGG CGT CTC TGA AAA AT	108
*RASA3*	GGA GAA GCT GGA GGA GGA GT	AAT GTC GAT CCA GTC CTT GG	118
*BRLF1*	ATG GCT GCT GCT TCC TTC TGG	CGA GGC AAG TCA TCT GTG G	108
*BGLF5*	GAG GAC ACG GTC AAG GAC AT	CTC CGG TCG GTG AAC AGT AT	167
*BZLF1*	GAC CCA TAC CAG GTG CCT TTT G	GCA CAC AAG GCA AAG GAG CTT G	97
*BORF2*	ACA CGC AGG GGG ATG AAC TCC T	ATG GAT TCT CGC TAG CCC GCC T	97
*BARF1*	ACC TGT GCC GCA TGA AAC TGG G	ACC TGA GCG TGG TGA AGC CTC T	70
*BXLF1*	AAA GAG AGC GGC CCA AGA	ACG ATT TGA CCT CAC ACG AG	111
*BALF5*	AGA AGG TCA CGC GCC GTT C	GTG CTT GTC TTG CAG CCA CG	119
*BMRF1*	ACC TGC CGT TGG ATC TTA GTG	GCG TTG TTG GAG TCC TGT G	128
*BBLF2/3*	GCT ATG GCA CCT CAG ACA	CCC AGG ATG AAC TCG TCT GC	124
*BALF2*	GGA CCC CTA TGT GAT CTC G	CAG GTT CTG GTT CAG CTG C	148
*BGLF4*	CGG TTT GAG CAC CCT CAT CT	GGC AAA CGT GTA GGA GGT CA	127
*BMLF1*	ACA CAT GGC TGG ATG CAC GCA T	GCA CCC TGG TGG CCA TGC TAG A	70

### qPCR to quantify cell-associated viral genomes and released viral genomes

Cell-associated EBV DNA was extracted and relative viral DNA levels were quantified through qPCR by targeting the EBV *BALF5* gene. To measure the relative amount of released EBV particles, equal volumes of culture supernatants were treated with DNase, and then qPCR was performed using primers specific to the EBV *BALF5* gene. The data was fit to a standard curve generated by amplifying the *BALF5* gene from 10-fold dilutions of the p2089 EBV bacmid used as template. The primers used to amplify EBV *BALF5* gene were previously published [97].

### Western blot analysis

Total cell extracts were electrophoresed in 8–12% SDS polyacrylamide gels and transferred to nitrocellulose membranes (Bio-Rad). Polyclonal antibodies were raised in rabbits against the EBV BGLF5 polypeptide (aa1–470) expressed in *E. coli* and purified by Ni++ affinity [4]. Rabbit antibodies were used to detect acetylated histone H3 (Upstate #06–599), EBV BALF5 protein (MyBioSource #MBS968779), PABPC4L (Abgent #16381b), β-actin (Abcam #ab8227), GAPDH (Abcam #ab8245), ZEBRA (S1605) [98], RTA (Sydney) [93], and VCA/FR3 (S1931) [99]. Mouse monoclonal antibodies were used to detect IL6 (R&D Systems #MAB206), FLAG (Sigma #F1804) β-actin (Sigma #A5316 or Sigma-Aldrich #A1978), ELAVL4/HuD (Novus # H00001996-M01 and Santa Cruz #sc-48421), EA-D (Millipore-Sigma #MAB8186), and α-Actinin (ThermoFisher #MA5–36095), followed by rabbit anti-mouse HRP (ZyMAX #81–6700) or goat anti-mouse HRP (ThermoFisher #626520). Antibody-protein complexes were detected using ^125^I-labeled protein A or ECL Plus (GE Healthcare).

### Statistical analysis

Student’s *t*-test was used to assess statistical significance when performing pair-wise comparisons. Results are expressed as the mean ± standard error of the mean (SEM) for experiments with three or more biological replicates or standard deviation (SD) for other experiments. *P* values, calculated using Microsoft Excel or GraphPad Prism5, are shown for experiments with at least 3 independent replicates.

## Supporting information

S1 Fig Separation of HH514–16 cells into lytic and refractory subpopulations. HH514–16 cells were treated with NaB for 48 hours. They were incubated with EBV antibody-positive human serum from a healthy donor and FITC-conjugated anti-human IgG. The cells were separated into lytic and refractory subpopulations using a FACSVantage cell sorter. Pre-sorted cells and gating strategy are depicted by the histogram and dotplot on the left while purity of post-sorted lytic and refractory cells is shown using histograms on the right.
(TIF)

S2 Fig BGLF5, the EBV protein that mediates host shut off, is selectively expressed in the lytic subpopulation of HH514–16 cells. HH514–16 cells were treated with NaB for 48 hours. Total cell extracts from control, refractory or lytic subpopulations were electrophoresed on an 8% SDS-PAGE gel and immunoblotted using indicated antibodies.
(TIF)

S3 Fig ELAVL4/HuD is induced upstream of viral genome replication in BL and LCL while *PABPC4L* is partially depending on replication in LCL. HH514–16 cells and LCL were treated with NaB, TPA, and PAA as indicated for 24 hours. Total RNA was isolated and *ELAVL4*, *PABPC4L*, and *BZLF1* transcripts were quantified using RT-qPCR (**A, C, and D**). Cell lysates were subjected to immunoblotting in **B** or analyzed by qPCR using primers targeting *BALF5* to quantify intracellular viral genomes in **E**. Representative of biological duplicates shown; error bars, SD of technical triplicates.
(TIF)

S4 Fig Expression of the EBV lytic cycle activator ZEBRA upregulates *ELAVL4* and *PABPC4L* transcripts only in EBV-infected cells. EBV-negative BJAB **(A)** and EBV-positive HH514–16 **(B)** cells were transfected with an empty vector (EV) or vector expressing *BZLF1* (p*BZLF1*). After 24 hours, cell extracts were analyzed by RT-qPCR using primers targeting *BZLF1*, *ELAVL4*, and *PABPC4L*. Error bars, SEM of 3 biological replicates, with 3 technical replicates within each biological replicate; ***, p < 0.001; ****, p < 0.0001.
(TIF)

S5 Fig Ectopic ELAVL4 and IL6 modestly suppress ZEBRA and RTA levels. As part of the experiments shown in Figs 6A and 7A, HH514–16 cells were transfected with empty vector CMV/RTS alongside vectors expressing *BZLF1* (Z), *BRLF1* (R), ELAVL4, IL6, or combinations as indicated. Lysates were harvested after 24 hours and subjected to immunoblotting using indicated antibodies.
(TIF)

S6 Fig shRNA-mediated depletion of ELAVL4 and PABPC4L transcripts suppresses lytic genome replication. HH514–16 cells and LCL were transduced with lentivirus for control shRNA, *ELAVL4* shRNA, or *PABPC4L* shRNA on two consecutive days. Twenty-four hours later, *ELAVL4* shRNA-transduced cells were exposed to lytic cycle triggers (NaB or NaB + TPA) and harvested after another 24 hours. For *PABPC4L* shRNA-exposed cells, cells were exposed to 10 days of puromycin selection starting at 24 hours after the second transduction. Cells were then washed and placed in puromycin-free medium for 24 hours, and then treated with lytic cycle triggers for another 24 hours. Cells were harvested and subjected to *BALF5* qPCR to quantify intracellular viral genomes (**A and B**), immunoblotting (**C**), and RT-qPCR analysis of *PABPC4L* transcripts (**D**). Representative of biological duplicates shown; error bars, SD of technical triplicates.
(TIF)

S1 Data Minimal anonymized dataset necessary to replicate the findings. (XLSX)
