# Supplementary figures and images for: Playing both sides – Epstein-Barr Virus accumulates select cellular transcripts to counter virus-mediated host shut-off in lytic cells
Source: PLoS Pathog. 2026 May 11;22(5):e1014211. doi: 10.1371/journal.ppat.1014211 (PMC13225664; doi:10.1371/journal.ppat.1014211)

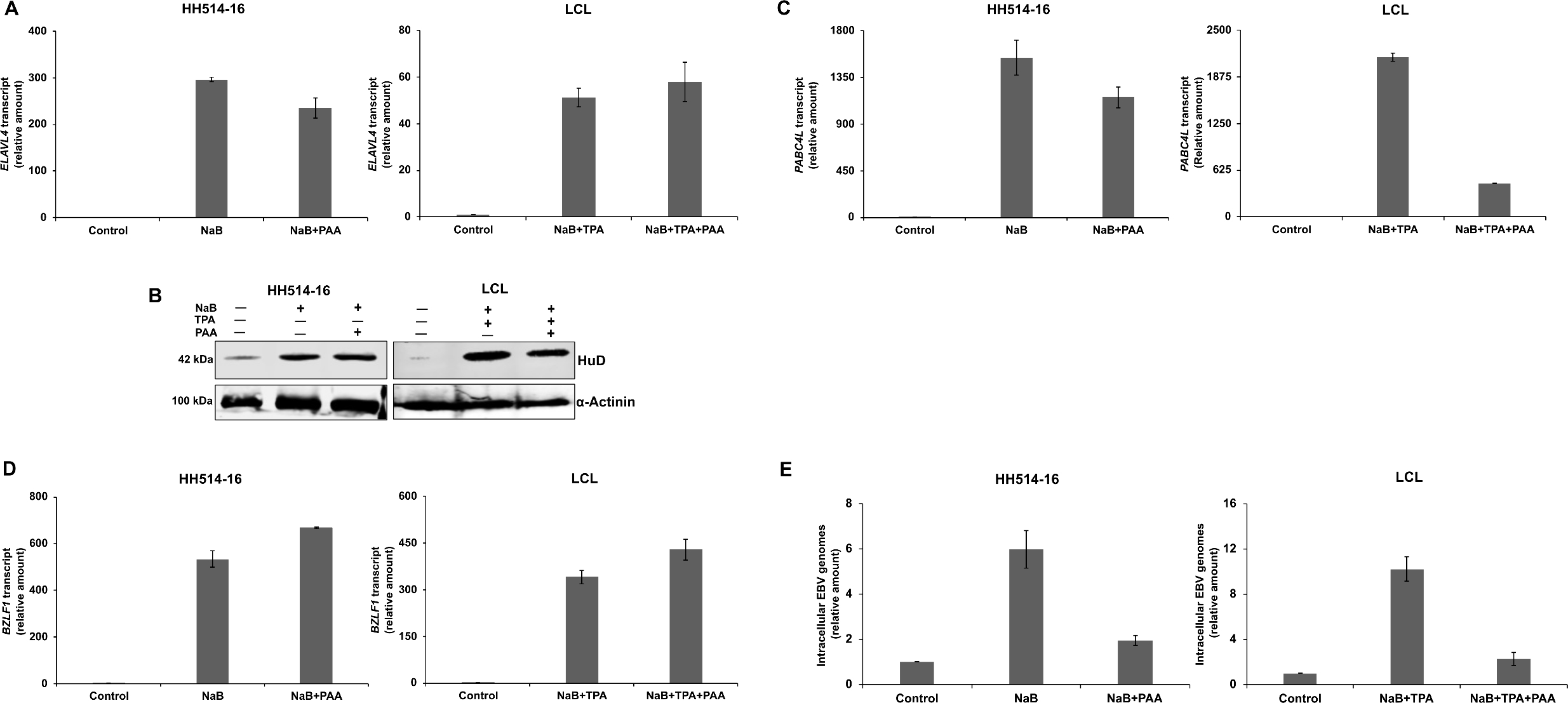

Supplement: S3 Fig — HH514–16 cells and LCL were treated with NaB, TPA, and PAA as indicated for 24 hours. Total RNA was isolated and ELAVL4, PABPC4L, and BZLF1 transcripts were quantified using RT-qPCR (A, C, and D). Cell lysates were subjected to immunoblotting in B or analyzed by qPCR using primers targeting BALF5 to quantify intracellular viral genomes in E. Representative of biological duplicates shown; error bars, SD of technical triplicates. (TIF) [file ppat.1014211.s003.tif]
